# A whole genome sequencing approach to anterior cruciate ligament rupture–a twin study in two unrelated families

**DOI:** 10.1371/journal.pone.0274354

**Published:** 2022-10-06

**Authors:** Daneil Feldmann, Christian D. Bope, Jon Patricios, Emile R. Chimusa, Malcolm Collins, Alison V. September

**Affiliations:** 1 Division of Physiological Sciences, Department of Human Biology, University of Cape Town, Cape Town, South Africa; 2 Department of Mathematics and Computer Science, Faculty of Sciences, University of Kinshasa, Kinshasa, Democratic Republic of Congo; 3 Division of Human Genetics, Department of Pathology, Faculty of Health Sciences, University of Cape Town, Cape Town, South Africa; 4 Centre for Bioinformatics, Department of Informatics, University of Oslo, Oslo, Norway; 5 Wits Sport and Health (WiSH), School of Clinical Medicine, Faculty of Health Sciences, University of the Witwatersrand, Johannesburg, South Africa; 6 Department of Applied Sciences, Faculty of Health and Life Sciences, Northumbria University, Newcastle, Tyne and Wear, United Kingdom; 7 Institute of Infectious Disease and Molecular Medicine, Faculty of Health Sciences, University of Cape Town, Cape Town, South Africa; 8 UCT Research Centre for Health Through Physical Activity, Lifestyle and Sport (HPALS), Cape Town, South Africa; 9 International Federation of Sports Medicine (FIMS) Collaborative Centre of Sports Medicine, Cape Town, South Africa; Government College University Faisalabad, PAKISTAN

## Abstract

Predisposition to anterior cruciate ligament (ACL) rupture is multi-factorial, with variation in the genome considered a key intrinsic risk factor. Most implicated loci have been identified from candidate gene-based approach using case-control association settings. Here, we leverage a hypothesis-free whole genome sequencing in two two unrelated families (Family A and B) each with twins with a history of recurrent ACL ruptures acquired playing rugby as their primary sport, aimed to elucidate biologically relevant function-altering variants and genetic modifiers in ACL rupture. Family A monozygotic twin males (Twin 1 and Twin 2) both sustained two unilateral non-contact ACL ruptures of the right limb while playing club level touch rugby. Their male sibling sustained a bilateral non-contact ACL rupture while playing rugby union was also recruited. The father had sustained a unilateral non-contact ACL rupture on the right limb while playing professional amateur level football and mother who had participated in dancing for over 10 years at a social level, with no previous ligament or tendon injuries were both recruited. Family B monozygotic twin males (Twin 3 and Twin 4) were recruited with Twin 3 who had sustained a unilateral non-contact ACL rupture of the right limb and Twin 4 sustained three non-contact ACL ruptures (two in right limb and one in left limb), both while playing provincial level rugby union. Their female sibling participated in karate and swimming activities; and mother in hockey (4 years) horse riding (15 years) and swimming, had both reported no previous history of ligament or tendon injury. Variants with potential deleterious, loss-of-function and pathogenic effects were prioritised. Identity by descent, molecular dynamic simulation and functional partner analyses were conducted. We identified, in all nine affected individuals, including twin sets, non-synonymous SNPs in three genes: *COL12A1* and *CATSPER2*, and *KCNJ12* that are commonly enriched for deleterious, loss-of-function mutations, and their dysfunctions are known to be involved in the development of chronic pain, and represent key therapeutic targets. Notably, using Identity By Decent (IBD) analyses a long shared identical sequence interval which included the LINC01250 gene, around the telomeric region of chromosome 2p25.3, was common between affected twins in both families, and an affected brother’. Overall gene sets were enriched in pathways relevant to ACL pathophysiology, including complement/coagulation cascades (p = 3.0e-7), purine metabolism (p = 6.0e-7) and mismatch repair (p = 6.9e-5) pathways. Highlighted, is that this study fills an important gap in knowledge by using a WGS approach, focusing on potential deleterious variants in two unrelated families with a historical record of ACL rupture; and providing new insights into the pathophysiology of ACL, by identifying gene sets that contribute to variability in ACL risk.

## Introduction

Globally, the burden of non-communicable diseases such as coronary heart disease, type 2 diabetes and cancer has escalated [1]. Along with other lifestyle factors, physical inactivity is a major risk factor contributing to the increased prevalence of these health conditions [2]. To mitigate the risk, there has been an increase in exercise participation among sedentary populations, and in parallel an increased incidence of musculoskeletal injuries [3]. One of the most common debilitating musculoskeletal injuries to affect the lower limb is rupture of the ACL [4]. The majority of ACL ruptures occur through non-contact mechanisms, when torsional or translational load exceeds the capacity of the ligament [5]. Given the potential high costs and significant clinical consequences of ACL ruptures, improved comprehension of the risk factors, aetiology, and the mechanism of injury is thus an important step in prevention strategies [6].

Multifactorial in nature [7] various extrinsic and intrinsic risk factors have been implicated in the susceptibility to ACL ruptures, with a growing body of evidence now supporting a genetic contribution [8]. Previous research implicating polymorphisms in candidate genes with ACL rupture susceptibility, have focussed primarily on case-control genetic-association studies [9]. Some of these loci map to genes encoding structural components such as collagen and proteoglycans, while others encode for angiogenesis associated signalling molecules, and regulators of the extracellular matrix. However, most of these studies have explored candidate genes in single populations, which are limited by small sample sizes, and hence underpowered to accurately detect associations, specifically with rare variants. Moreover, regions of the genome with potential risk-modulating effects in other protein-coding regions, as well as deep intronic variants in non-coding regions of the genome, may have been overlooked using this candidate gene approach.

Magnusson [10] recently estimated a ~69% heritability component to ACL ruptures and it seems, a large heritability component remains unexplored. Technologies such as genome wide association studies and next generation sequencing (NGS) both hold a promise to increase our understanding of the genetics underlying ACL rupture and to identify new genetic loci for future investigation, which in turn will assist in elucidating the molecular mechanism of injury. Genome wide association studies have to date largely used canine models [11–13] with two human studies highlighting three independent polymorphisms to be associated with ACL rupture but with borderline significance [14] and recently three additional novel loci that met genome significance to associate with ACL and PCL injury [15]. Although the biological effect and their clinical significance still need to be determined, a whole exome sequencing strategy was used to identify 11 novel variants that associate with ACL rupture in a twin family study [16]. There is still much paucity in the application of NGS technologies to identify genetic loci for ACL rupture susceptibility.

To build on previous research, the current study aimed to apply a whole genome sequencing approach (WGS) to two unrelated families each with twins with a history of recurrent ACL ruptures acquired playing rugby as their primary sport. The aim is to identify novel or previously implicated biological genomic signatures, which can be explored to further characterise ACL rupture susceptibility. The main objectives of the study are to (i) identify predicted pathogenic or potential modifiable variants for ACL rupture susceptibility, and (ii) identify genetic sequences or genetic intervals common between affected members within families or affected members between families. This study fills an important gap, and adds to the growing knowledge of the natural history, and clinical heterogeneity of ACL rupture, with the potential for informing the design of new therapeutics.

## Results

### Participant characteristics and clinical descriptors

**Fig 1:**
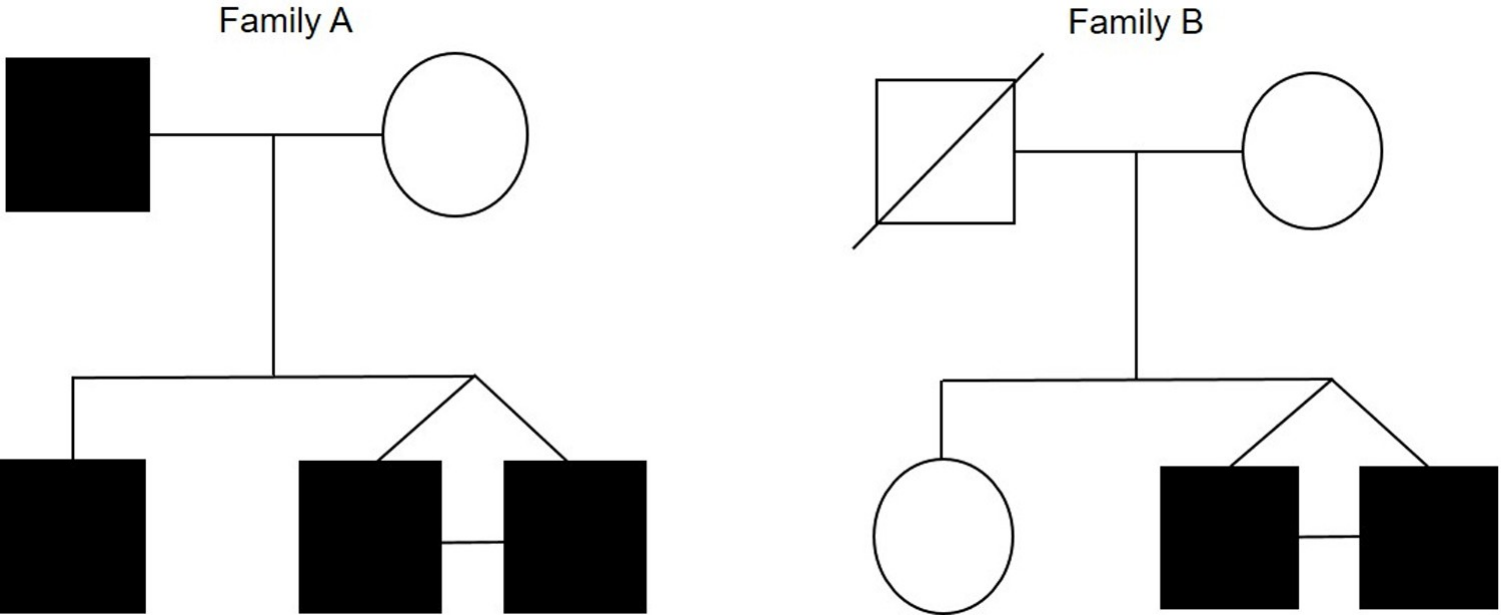
Pedigree structure for Family A and Family B with known history of surgically diagnosed non-contact anterior cruciate ligament (ACL) rupture. Circles indicate females, squares, males. Filled symbols indicate participants surgically diagnosed with non-contact anterior cruciate ligament rupture, and open symbols uninjured participants. Symbols with a line through them indicate deceased individuals.

Two unrelated twin families with a family history of ligament and other musculoskeletal soft tissue (**S1 Table**) were recruited from South African families of Dutch/Irish (Family A) and English/Scottish (Family B) descent, respectively (**Materials and Methods**). The pedigrees of both families are summarised in **Fig 1**.

Family A monozygotic twin males (Twin 1 and Twin 2) were recruited at 33 years old. Both individuals sustained two unilateral non-contact ACL ruptures of the right limb. Twin 1 at age 20 and 27 years, and Twin 2 at age 28 and 32 years (**Fig 1**). All ACL ruptures occurred while playing club level touch rugby, and the number of years of participation were 17 years and 12 years for Twin 1 and 2, respectively. Both individuals had a previous history of other ligament injuries (**S1 Table**). Twin 1 had sustained injuries to the lateral ligament of the right ankle, and the extensor tendon of the wrist. Twin 2 had sustained an injury to the medial ligament of the right ankle. Their male sibling was recruited at 41 years old, and had sustained bilateral non-contact ACL ruptures at 12 and 31 years of age, both while playing rugby union. The number of collective years of participation was 10 years, and the sibling also had a previous history of bilateral injury to the tibialis posterior tendon. The father, recruited at 61 years, had sustained a unilateral non-contact ACL rupture on the right limb at the age of 30 years. The rupture occurred while playing professional amateur level football (38 years of participation), however, no history of previous other ligament or tendon injuries were recorded. The mother, recruited at age 58, reported participating in dancing for over 10 years at a social level, with no previous ligament or tendon injuries (**S1 Table**).

Family B monozygotic twin males (Twin 3 and Twin 4) were recruited at the age of 29 years. Twin 3 had sustained a unilateral non-contact ACL rupture of the right limb at the age of 27 years, and Twin 4 sustained three non-contact ACL ruptures (two in right limb and one in left limb) at ages 21, 25, and 27 years (**Fig 1**). Both individuals ruptured their ACL playing provincial level rugby union, which they had participated in for 10 years, and both reported a history of previous another ligament or tendon injury (**S1 Table**). Twin 3 had sustained injuries to the lateral ligament of the right ankle, the right shoulder ligaments and the supraspinatus tendon of the right shoulder. Twin 4 had sustained an injury to the medial ligament of the right ankle. Their female sibling and mother (31 and 58 years at recruitment respectively) had no previous history of ligament or tendon injury. The female sibling had participated in karate and swimming activities (10 and 26 years of participation respectively) and the mother in hockey (4 years) horse riding (15 years) and swimming (8 years of participation). The father was deceased at the time of recruitment, and a medical history was therefore unavailable. However, the family reported no known history of any ligament or tendon injury.

### Variant discovery and quality control

A total number of 10,560,616 variants were called in the whole genome sequence dataset, of which 1.13% were exonic (distributed as 5,2% nonframeshift deletion, 3% nonframeshift insertion, 19% frameshift deletion, 14% frameshift insertion, 31% nonsynonymous, 24% synonymous, 3% stopgain, 0,08% stoploss and 1.1% unknown), 0.35% ncRNA_exonic, 53% intergenic, 43% intronic, 0.03% splicing, 0.96% UTR3, 0.12% UTR5, 0.64% upstream, 0.66% downstream and 0.46% other. The overall quality control of the generated data can be found in **S1 Fig**.

### Genetic structure

**Fig 2:**
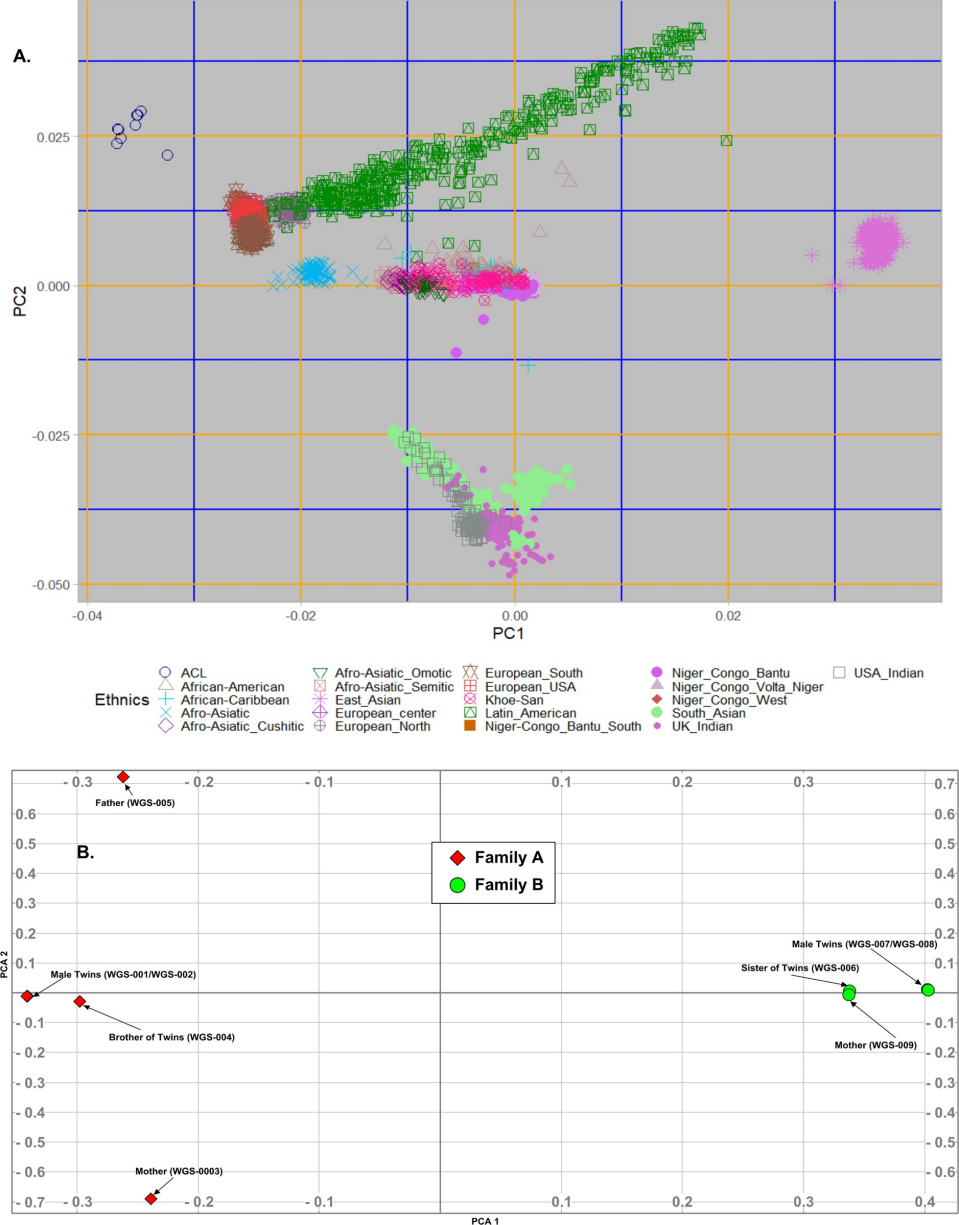
Principal component analysis on merged data sets of Family A and B. Family A is depicted in red diamonds, with Family B in green circles.

From **Fig 2A**, we observed that South African families of Dutch/Irish (Family A) and English/Scottish (Family B) descent formed a cluster close to European’s Cluster. This separation justifies that both ACL families are the result of genetic drift in an isolated founder population, that occurred with the Euro-Asia settlement in South Africa since 1652 [17].

Principal component analysis (PCA) of data from Families A and B, showed that the two families (**Fig 2B)** clustered separately from each other. Within Family A, the twins were very close together, with their male sibling in close proximity. However, the father and mother positioned further away from all the progenitors. The individuals in Family B on the other hand, were more closely grouped, with the uninjured mother and female sibling in close proximity to the twins.

### In silico putative deleterious variants

A total number of 10,560,616 variants were called in the whole genome sequence dataset and therefore we needed to prioritise these variants and their implicated genes based on which variants are most likely deleterious using various bioinformatic tools. These identified candidate genes maybe clinically relevant and assist in unravelling the aetiology of ACL injury risk and assist in identifying potential clinically relevant therapeutic targets for ACL injuries. Filtering mutations, 29 genes with predicted mutations were prioritized for Family A (**S2 Table**) of which variants within two genes, namely *COL11A1* (ENSG00000060718) and *COL12A1* (ENSG00000111799) which encode for the α1 chains of types XII and XI collagen respectively, have previously been associated with ACL rupture. For Family B, a list of 18 genes, including the previously associated *COL12A1*, were prioritized (**S2 Table**). Of the 47 genes prioritized (29 in Family A, and 18 in Family B) three genes *COL12A1*, *CATSPER2* (ENSG00000166762) and *KCNJ12* (ENSG00000184185), were common to both families (**Table 1**). *CATSPER2* and *KCNJ12*, which have not previously been investigated as candidate genes, both encode for ion channels associated with cation and potassium ion channels, respectively. Six non-synonymous SNPs were identified within these three genes, one each in *COL12A1* and *CATSPER2*, and four in *KCNJ12* (**Table 1**). From SIFT, PolyPhen-2 and FATHMM_pred prediction tools, the Gly3058Ser (ENSP00000325146.8) substitution at *COL12A1* rs970547 C>T (NC_000006.12) and the Arg511His (ENSP00000321463.5) substitution at *CATSPER2* rs144399798 C>T (NC_000015.10) were predicted moderately damaging (CADD 20–30) with deleterious loss of function effect. While the Glu139Lys (ENSP00000328150.5), Gly145Ser (ENSP00000328150.5), Arg261His (ENSP00000328150.5), and Ile262Ser (ENSP00000328150.5) substitutions at *KCNJ12* rs76265595 (NC_000017.11), rs75029097 (NC_000017.11), rs77270326 (NC_000017.11) and rs76684759 (NC_000017.11) variants, respectively, were predicted strongly damaging (CADD > 30) with deleterious loss of function effect. The glycine to serine substitutions at rs970547 and rs75029097 result in a change from a non-polar, aliphatic to a polar non-charged amino acid. For the arginine to histidine substitutions at rs144399798 and rs77270326, a basic polar amino acid is substituted for another basic polar amino acid, whereas for the glutamate to lysine substitution at rs76265595, an acidic polar amino acid is substituted for a basic amino acid. Lastly, the isoleucine to serine substitution at rs76684759, results in a non-polar, aliphatic amino acid change to a polar non-charged residue [18].

**Table 1:** Predicted functional effect of mutations common to Family A and Family B.

Number of Patients	Genotype	Gene	Region	SNP	Protein Change	Functional effect	ExAC AFR	ExAC EUR
(9)	Homozygous	*COL12A1* (ENSG00000111799)	6q14.1	rs970547 C>T (NC_000006.12)	Gly3058Ser (ENSP00000325146.8)	Loss of function of growth plate cartilage chondrocyte morphogenesis	0.0026	0.73
(9)	Homozygous	*CATSPER2* (ENSG00000166762)	15q15.3	rs144399798 C>T (NC_000015.10)	Arg511His (ENSP00000321463.5)	Loss of function in ion channel activity and voltage-gated ion channel activity.	0.0083	0.0003
(9)	Homozygous	*KCNJ12* (ENSG00000184185)	17p11.2	rs76265595 G>A (NC_000017.11)	Glu139Lys (ENSP00000328150.5)	Loss of function of inward-rectifier potassium channels	0	0.71
				rs75029097 G>A (NC_000017.11)	Gly145Ser (ENSP00000328150.5)	Loss of function of inward-rectifier potassium channels	0	0.32
				rs77270326 G>A (NC_000017.11)	Arg261His (ENSP00000328150.5)	Loss of function of inward-rectifier potassium channels	0.001	0.11
				rs76684759 T>G (NC_000017.11)	Ile262Ser (ENSP00000328150.5)	Loss of function of inward-rectifier potassium channels	0.002	0.017

### 3D protein structure analysis

**Fig 3:**
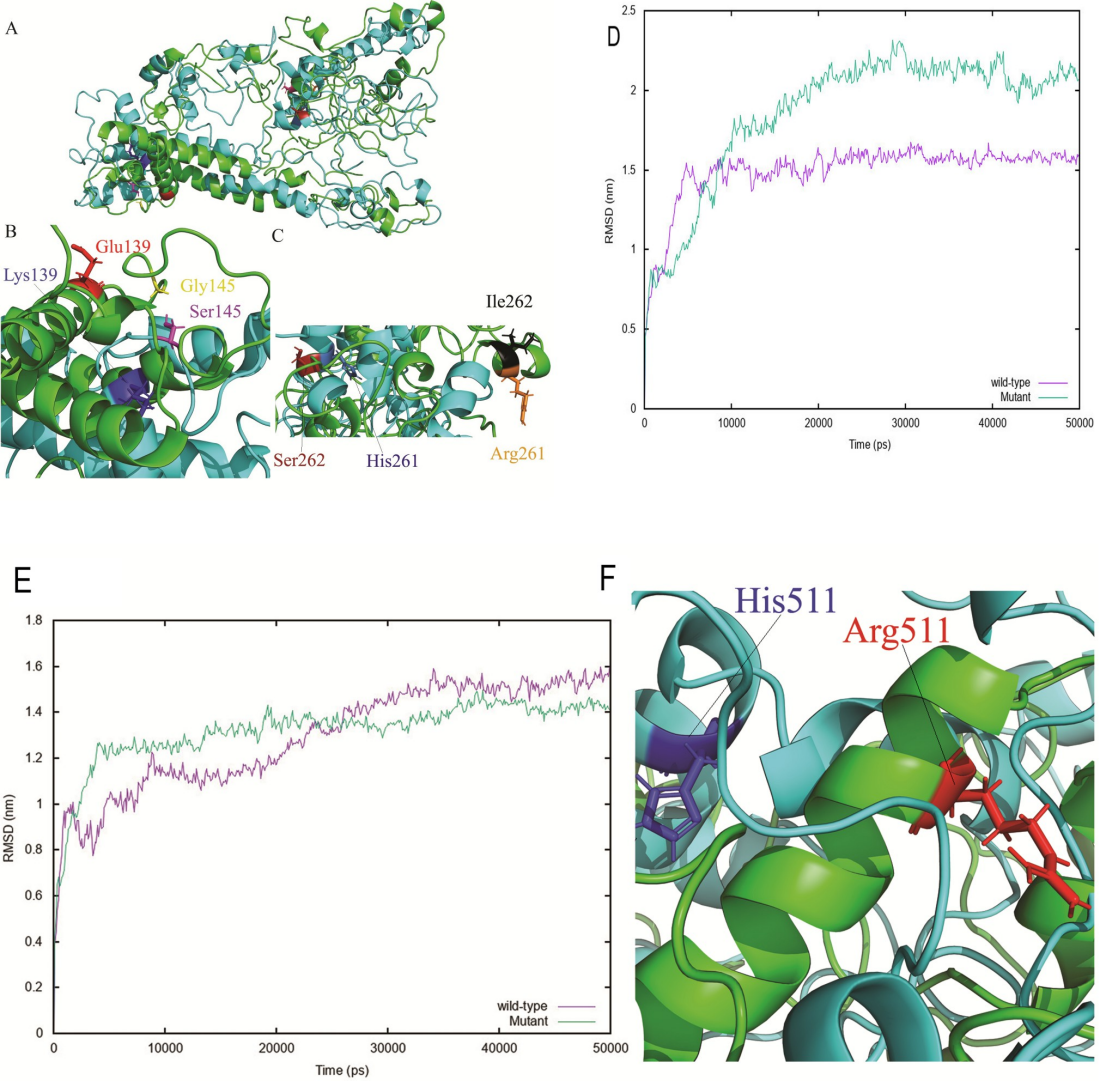
Molecular dynamic simulations for *KCNJ12* and *CATSPER2*. A: 3D protein structure of mutations in *KCNJ12* comparing the wild type (red) and mutant (blue) forms. B: *KCNJ12* residue change at Glu139 (red) to Lys139 (blue) and Gly145 (yellow) to Ser145 (pink). C: *KNCJ12* residue change at Arg261 (orange) to His261 (skyblue), and Ile261 (black) to Ser262 (firebrick). D: Root mean square displacement of all *KCNJ12* atoms in wild type (purple) and mutant form (green). E: Root mean square displacement of all *CATSPER2* atoms in wild type (purple) and mutant form (green), and F: *CATSPER2* residue change at Arg511 (red) to His511 (blue).

Focusing on the two novel candidate genes, we have conducted a simulation of 3D protein structure (**See** **Materials and Methods**) and molecular dynamic simulation results for *KCNJ12* and *CATSPER2* are shown in **Fig 3**. The 3D structure displays the four residue mutations within the *KCJN12* protein; mutation of the polar uncharged Glu139 to the hydrophilic positively charged Lys139; the non-polar Gly145 to the hydrophilic polar non-charged Ser145, the positively charged Arg261 to the positively charged His261; and the non-polar Ile262 to the polar uncharged and hydrophilic Ser262 which contribute to a more flexible mutated structure. The flexibility of the structure may impact binding interactions, stability, and conformation of the protein. **Fig 3A** shows the 3D structure superposition of *KCJN12*, comparing the wild type (red) and mutant (blue) illustrating the flexibility of the structures. **Fig 3B** highlights the residue change from Glu139 (red) to mutated residue Lys139 (blue) and Gly145 (yellow) to residue Ser145 (pink). **Fig 3C** highlights the residue changed from Arg261 (orange) to mutated residue His261 (skyblue) and residue Ile261 (black) to residue Ser262 (firebrick). **Fig 3D** displays the Root Mean Square Displacement (RMSD) of all atoms within the *KCJN12* wild-type (pink) and with mutated residues (green). **Fig 3E** shows the RMSD of all atoms in the *CATSPER2* wild-type (pink) and with mutated residues (green) and **Fig 3F** displays the structure of the positively charged Arg511 residue (red) mutated to the positively charged His511 residue (blue) illustrating the rigidity of the mutated structures. **S2 Fig** shows the 3D structure superposition of *CATSPER2* protein site, comparing the wild type (green) and mutant (cyan) forms.

### Pathways and biological processes associated with genes with mutational burdens

**Fig 4:**
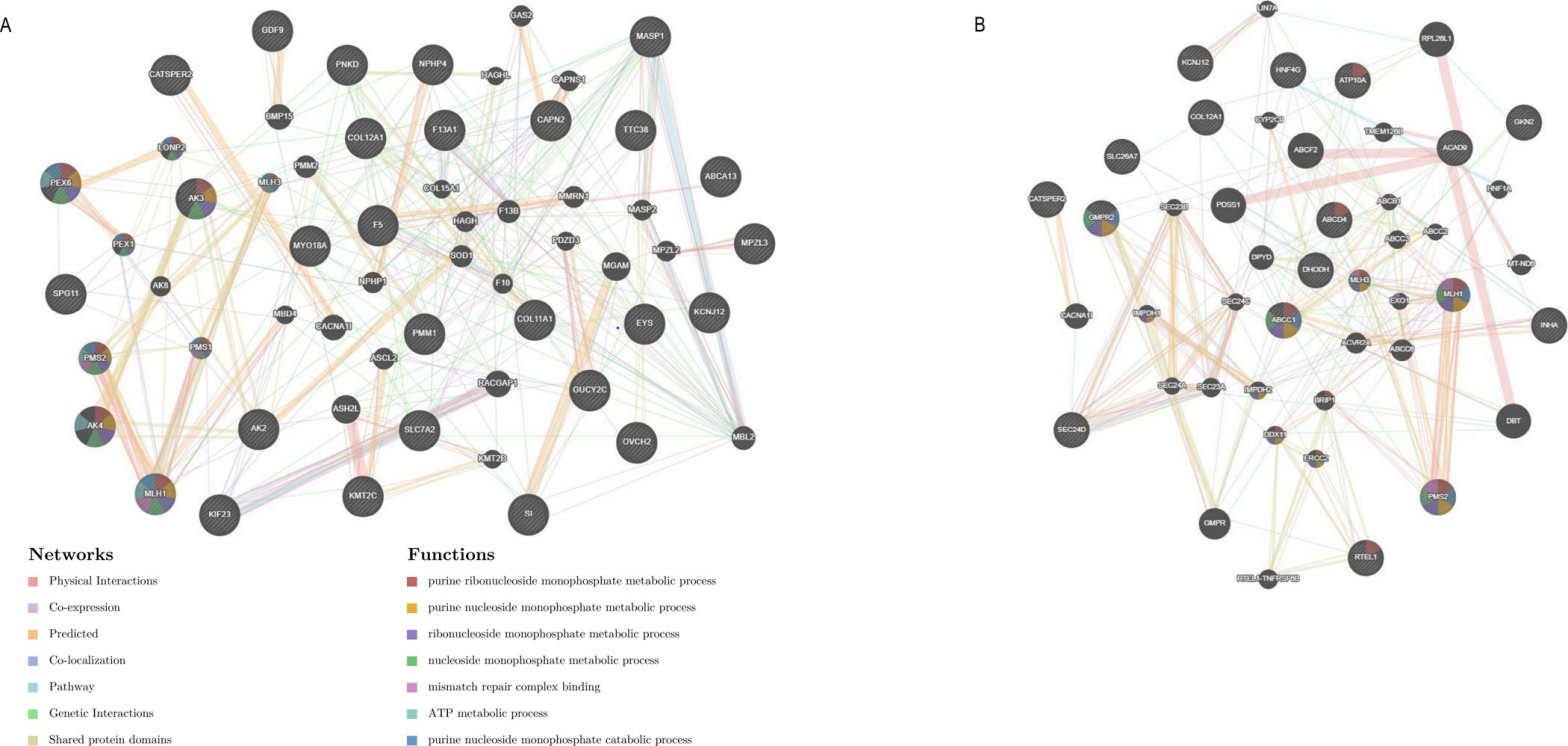
A: Gene-specific proportion of pathogenic SNPs across 20 ethnic groups, and Family A and B from 40 genes known to associate with tendon and ligament injury and B: Gene-specific SNPs minor allele frequencies across 20 ethnic groups from 40 genes known to associate with tendon and ligament injury.

To determine potential interactions and functional pathways for the prioritized candidate genes in **Table 1**, an interaction network between the predicted pathogenic and genetic modifier variants for Family A and B was generated (**Fig 4A and 4B**). Physical interactions, co-expression, predicted, co-localization, pathways and genetic interactions are depicted in the **Fig 4**. Furthermore, functions of the genes in the networks are distinguished by colour coding and classified according to metabolic process. Candidate gene sets (**S2 Table** and **Fig 4**) were enriched in pathways relevant to ACL pathophysiology, including *complement/coagulation cascades* (p = 3.0e-7), *purine metabolism* (p = 6.0e-7) and *mismatch repair* (p = 6.9e-5) pathways. Gene-set previously associated with ligament and tendon injury (**S3 Table**) were enriched in pathways including *PI3K-Akt signalling pathway* (p = 8.9e-11), *protein digestion and absorption* (p = 5.9e-10) and *rheumatoid arthritis* (p = 1.1e-6).

Additional enriched top significant (p < 0.05) pathways, biological processes, molecular function, and human phenotypes associated with the genes previously associated with ligament and tendon injury (**S3 Table**) and the candidate list of predicted pathogenic genes and their interacting genes for Family A and Family B (**S2 Table and Fig 4**, respectively) are shown in **S4 Table**.

### Distribution of pathogenic variants, minor allele and gene-specific in SNP frequencies

**Fig 5:**
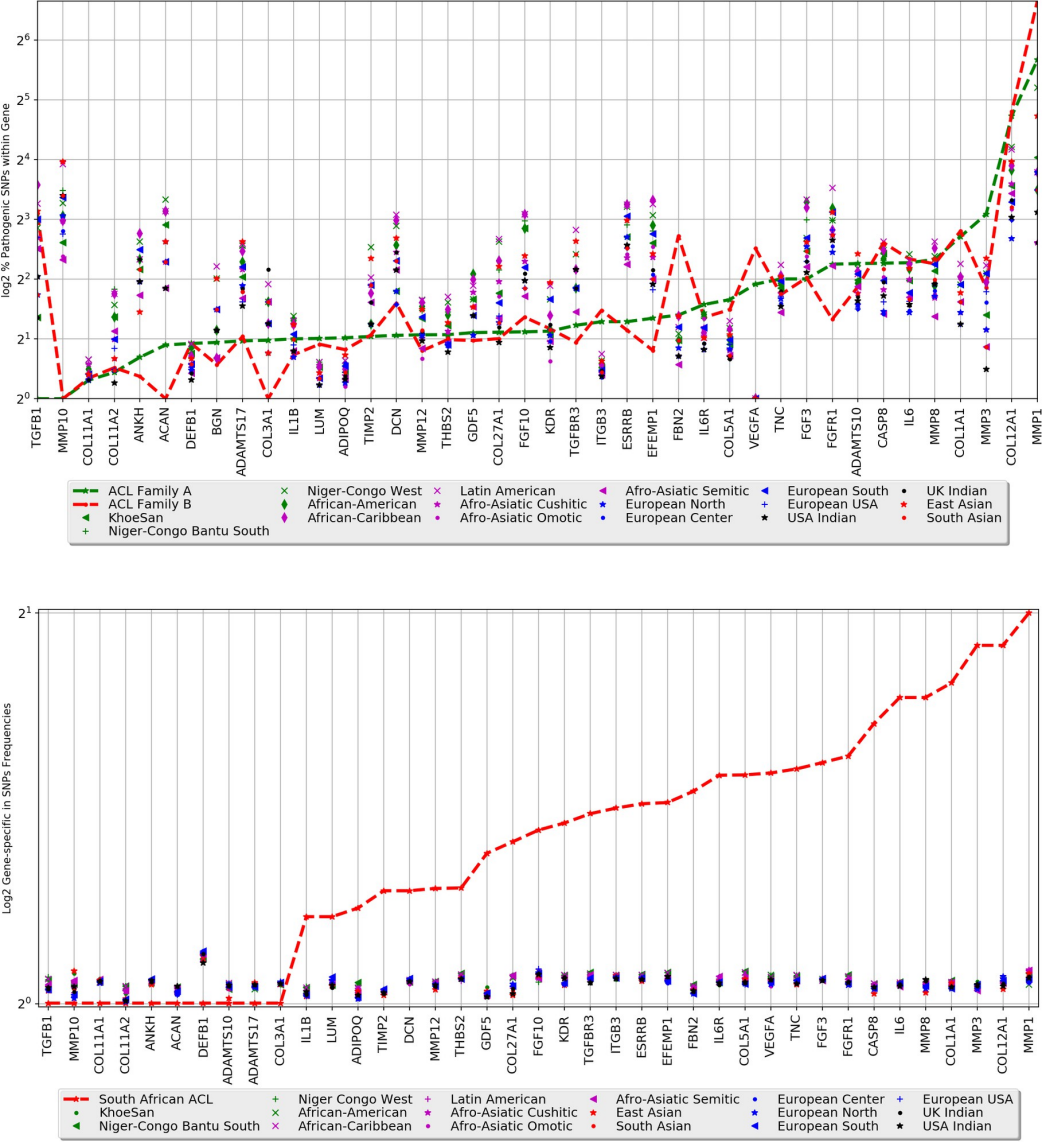
Biological sub-network of the candidate mutant genes with interacting genes in Family A (A) and Family B (B). Candidate genes common to Family A and B are highlighted by red stars. Weighting of interaction is depicted by line thickness. All query genes are given the maximum node size, and the size of related genes is inversely proportional to the rank of the gene based on its score assessed by GeneMANIA [19].

We examined the distribution of reported pathogenic variants across worldwide ethnics and our family dataset, in genes previously associated with risk of ligament and tendon injuries. *ADIPOQ* (ENSG00000181092), *COL5A1* (ENSG00000130635), *COL11A1*, *COL12A1*, *DEFB1* (ENSG00000164825), *FBN2* (ENSG00000138829), *ITGB3* (ENSG00000259207), *LUM* (ENSG00000139329), *MMP1* (ENSG00000196611) and *VEGFA* (ENSG00000112715) had a considerable higher proportion of pathogenic SNPs in both families compared to 20 other ethnic groups (**Fig 5A**). Moreover, for the *TGFB1* (ENSG00000105329), *FBN2*, *VEGFA* and *MMP1* genes, Family B had a higher proportion of pathogenic SNPs compared to 20 other ethnic groups, and Family A (**Fig 5A**). Of these, *COL11A1* was prioritised with predicted mutations in Family A, and *COL12A1* with predicted mutations in both families (**S2 Table**). Of the 40 genes previously associated with ligament and tendon injury, 29 genes had a greater gene-specific SNP frequency when compared to the other 20 ethnic groups (**Fig 5B**). Notably, *CASP8* (ENSG00000064012), *IL6* (ENSG00000136244), *MMP8* (ENSG00000118113), *COL1A1* (ENSG00000108821), *MMP3* (ENSG00000149968), *COL12A1* and *MMP1* genes had the highest gene-specific SNP frequency of > 1.6. Again, the previously prioritised *COL12A1*, was highlighted as one of the genes with the highest gene-specific SNP frequency (**Fig 5B**).

Furthermore, we observed variation of the distribution of MAF at rare and common variants (**S3 Fig)** between the South African families of Dutch/Irish (Family A) and English/Scottish (Family B) descent and the rest of 20 worldwide ethnic groups. The South African families of Dutch/Irish (Family A) and English/Scottish (Family B) have a lower frequency of rare variants at MAF between 0.0 and 0.1, and higher frequency of common variants at MAF bin 0.1–0.5 in contrast to those from 20 ethnic groups (**S3 Fig**).

### Shared IBD, functional partners, and further enrichment analyses

Identity by descent (IBD) analyses is used in family studies to identify shared genetic signatures between affected siblings as a mapping strategy to identify plausible genetic regions to harbour disease-causing mutations. We therefore used this approach as part of our strategy to identify genetic intervals shared between affected members within a family and between families. Each of the tools used in this study have limitations and we tried to combine the strengths to prioritise a list of genes and variants to explore. The IBD analyses highlights identical sequence stretches overlapping several genes. It does not target a specific polymorphism. We hereby leveraged IBD analyses to explore sequence regions of the genome common (identical) within and between families, we identified 17 genes in three sequence regions that were shared and identical between Twin 1 and Twin 2 of Family A (**Table 2**). In addition, both Family A twins shared the identical sequence for *LINC01250* (ENSG00000234423) gene on chromosome 2p25.3, which encodes for long intergenic non-protein coding RNA 1250, with their affected brother. The affected father and affected brother shared an identical sequence segment on chromosome 14q32.33, which consisted of 29 genes, and the unaffected mother shared 20 genes with the affected brother in a segment located at 19q13.42. For Family B, twin males shared four identical segments comprising a total of 37 genes (**Table 2**) and Twin 3 shared an identical gene segment on chromosome 13q34 which included one gene, with the unaffected sister. In addition, Family A twins, their affected brother and Family B twins also shared the identical sequence for *LINC01250* gene located on chromosome 2q25.3. And common to both families, both sets of twins shared the identical sequence for the 15q11.2 region comprising 8 genes, and the unaffected mother and affected brother from Family A shared the identical sequence for the *KIR3DX1* (ENSG00000104970) gene with Twin 3 and 4 from Family B.

**Table 2:** Detecting shared identity-by-descent (IBD) sequence segments between family members in Family A and Family B.

Sample 1	Sample 2	Chromosomal Location	Start	End	Genes	LOD	IBD Segment Length
**FAMILY A**							
Twin 1 and Twin 2 (affected)	Brother (affected)	2p25.3	3005297	3057952	***LINC01250***	5.34	3.944
Twin 1 (affected)	Twin 2 (affected)	2q25.3	3005007	3058005	LINC01250*	6.37	3.945
		15q11.2	22292484	22584320	GOLGA6L22*, GOLGA8EP*, HERC2P2*, HERC2P7*, RN7SL545P*, SPATA31E3P*	5.94	3.59
		21q21.1	16448783	16633125	*MIR125B2*, *MIR99A*, *MIRLET7C*, *MIR99AHG*	10.67	2.077
Father (affected)	Brother (affected)	14q32.33	105952630	106208389	*ADAM6*, *IGHV1-2*, *IGHVIII-2-1*, *IGHV1-3*, *IGHV4-4*, *IGHV7-4-1*, *IGHV2-5*, *IGHVIII-5-1*, *IGHVIII-5-2*, *IGHV3-6*, *IGHV3-7*, *IGHV3-64D*, *IGHV5-10-1*, *IGHV3-11*, *IGHVIII-11-1*, *IGHV1-12*, *IGHV3-13*, *IGHVIII-13-1*, *IGHV1-14*, *IGHV3-15*, *IGHVII-15-1*, *IGHV3-16*, *IGHVIII-16-1*, *IGHV1-17*, *IGHV1-18*, *IGHV3-19*, *SLC20A1P2*	56.56	1.516
Mother (unaffected)	Brother (affected)	19q13.42	54184163	54537844	*CDC42EP5*, KIR3DX1*, *LAIR1*, *LAIR2*, *LENG8*, *LENG8-AS1*, *LENG9*, *LILRA4*, *LILRA5*, *LILRA6*, *LILRB2*, *LILRB3*, *LILRB5*, *MBOAT7*, *MIR4752*, *RNU6-1307P*, *RPS9*, *TSEN34*, *TTYH1*, *VN1R104P*	7.76	1.778
**FAMILY B**							
Twin 3 (affected)	Twin 4 (affected)	2q25.3	3009692	3047881	LINC01250*	3.31	2.748
		15q11.2	22370898	22524432	GOLGA6L22*, GOLGA8EP*, HERC2P2*, HERC2P7*, RN7SL545P*, SPATA31E3P*	4.3	1.918
		19q13.42	54529431	55093392	*EPS8L1*, *FCAR*, *GP6*, *GP6-AS1*, *KIR2DL1*, *KIR2DL3*, *KIR2DL4*, *KIR2DP1*, *KIR2DS4*, *KIR3DL1*, *KIR3DL2*, *KIR3DL3*, *KIR3DP1*, KIR3DX1*, *LILRA1*, *LILRA2*,	7.4	1.775
		19q13.42	54539272	55088813	*LILRB1*, *LILRB1-AS1*, *LILRB4*, *LILRP1*, *LILRP1*, *LILRP2*, *MIR8061*, *NCR1*, *NLRP2*, *NLRP7*, *PPP1R12C*, *RDH13*, *RNU6-222P*, *VN1R105P*	7.35	1.751
Twin 3 (affected)	Sister (unaffected)	13q34	111663671	111916421	*LINC00354*	4.31	1.551

**Bold** depicts genes common within affected members between families. * depicts genes common within (affected/unaffected) members between families.

From our prioritised list of genes with mutational burdens, and IBD analysis, inferred functional partners of selected genes of interest were explored. Genes (*COL11A1*, *COL12A1*, *CATSPER2*, *KCNJ12*, *GP6* (ENSG00000088053), *MIR99A* (ENSG00000207638), *MIR99AHG* (ENSG00000215386), *MIR125B2* (ENSG00000207863), *MIRLET7C* (ENSG00000199030) and *LINC01250*) were selected based on potential pathogenicity for ACL rupture, shared regions in affected family members, previously published associated genes, and shared biology/function (**S5 Table**). The majority of inferred functional partners identified for Family A and Family B genes of interest included *collagens*, *proteoglycans*, *glycoproteins*, *integrins*, *laminins*, *growth factors*, *interleukins*, *microRNAs*, *apoptotic genes*, *and protein kinases* (**S5 Table**). A few genes were implicated in ion channel activity and signalling, with others involved in reproduction and fertilization pathways. However, the large majority were implicated in regulating the synthesis and degradation of the components of the extracellular matrix, collagen fibrillogenesis and new blood vessel formation through angiogenesis related pathways (**S5 Table**).

## Discussion

Through the employment of a whole genome sequencing approach and a study design including individuals from two unrelated twin families, with a history of ACL injury. The findings presented in this study addresses potential function-altering variants and genetic modifiers in ACL rupture susceptibility. The main findings include (i) the identification of polymorphisms in three genes (*COL12A1*, *CATSPER2*, *KCNJ12*) that are commonly enriched for deleterious and loss-of-function mutations in a phenotypically defined family of patients, and with evidence of genetic association with different phenotypes (**Table 1 and S2 Table**), providing support for the complexity of the genetic architecture of ACL rupture phenotypic variability. In addition, (ii) a shared IBD segment including the *LINC01250* gene in the telomeric region of chromosome 2p25.3 was noted between affected twins in both families, and an affected brother (**Table 2**). Type XII collagen (*COL12A1*) is a fibril-associated collagen belonging to the interrupted triple helices (FACITs) family. In addition to its role in fibrillogenesis [20, 21], it provides a molecular bridge between fibrillar collagens, and other matrix molecules facilitating fibril interaction with other extracellular and cell surface molecules within ligaments [22]. It is interesting to note that *COL12A1* was one of the genes highlighted to have a higher SNP burden in the two twin families, compared to world populations (**Fig 5A**). Additionally, several case-control genetic association studies have previously explored the *COL12A1* rs970547 polymorphism identified in this study, with ACL rupture risk susceptibility [23–27].

The cation channel sperm associated 2 (*CATSPER2*) and potassium inwardly rectifying channel subfamily J member 12 (*KCNJ12*) genes have not been implicated with risk of susceptibility to ACL rupture, and are therefore novel and noteworthy. Ion channels within human tissues are ubiquitous, and channel defects are implicated in a wide variety of diseases affecting the nervous, cardiovascular, respiratory, endocrine, urinary system and immune systems [28]. Interestingly, 3D protein structure simulation analysis (**Fig 3 and S2 Fig**) illustrates the rigidity of the mutated structures for these potassium and ion-channel related genes, potential mutations have an impact binding interactions, stability, and conformation of these proteins. Also these potassium and ion-channel related genes, and their dysfunction have been implicated in the development of chronic pain conditions [29, 30] and subsequently identified as potential therapeutic targets for painful conditions [31]. Even more, ion channels modulate membrane ion conductance across all cells and tissues, establishing electrical fields that affect cellular behaviours under normal conditions, during critical periods of development, and in response to tissue injury [32]. Further to that, ion channels and transporters are directly involved in the angiogenesis pathway, as they are expressed by vascular endothelial cells [33] and are thought to contribute to vasodilation, in response to extracellular K+ concentration [34]. Therefore, the modified expression and activity of ion channels is likely related to vascular alteration in pathological conditions [31].

The angiogenesis related pathway plays a significant role in regulating ECM homeostasis, and perturbations of the expression of specific genes functioning in this pathway have also been implicated in contributing to the outcomes of surgical interventions such as ACL reconstruction [35]. These genes therefore represent new gene targets to explore the clinical heterogeneity and pathogenesis of ACL rupture, or in potential drug targeting strategies. New ideas for analgesic drug design are urgently needed, especially given the number of recent high-profile failures with some prospective targets (i.e. the neurokinin receptor 1 antagonists) [36, 37] which have caused many leading pharmaceutical companies to curb their focus in this area.

No shared IBD segments were identified at the regions where *COL12A1*, *CATSPER2* and *KCNJ12* are located, suggesting these mutations may not have occurred since the time of the most recent common ancestors and are therefore not founder mutations. Interestingly population structure analysis revealed Family A and Family B clustered separately, but in close proximity to the European’s cluster, possibly as a result of genetic drift in an isolated founder population, that occurred with the Euro-Asia settlement in South Africa since 1652 [17].

Interestingly, the two families shared an IBD segment that included a long intergenic non-protein coding RNA (lincRNA) *LINC01250* gene in the telomeric region of chromosome 2p25.3. It is known that the telomeric regions of any human chromosome harbour structural variants and repetitive nucleotide sequences [38] which was further illustrated by the high LD pattern among variants within non-protein genes, such as *LINC01250* (**S4 Fig**). LincRNAs function broadly to fine tune target gene expression by the direct modulation of nuclear architecture, in addition to indirectly through transcription or translation activities [39]. From this observation, one can hypothesize that structural variation and changes within the *LINC01250* region may contribute to the severity and phenotypic variation of ACL injury through the modulation of functional genes involved in ligament biology. Further investigation is needed to test this.

Notably, enriched pathways represented by our genes of interest point to relevant pathophysiological mechanisms affecting collagen fibrillogenesis, cell to cell communication, angiogenesis signalling, and homeostasis of the ECM through proteins such as integrins, interleukins, growth factors, glycoproteins and protein kinases (**S5 Table**) of which some are already therapeutic targets. Interestingly, recent evidence suggests that long noncoding RNA encoding genes are critical in angiogenesis and cell migration and proliferation pathways, with some lncRNA encoding genes vital to wound healing processes [40]. Furthermore, *COL1A1*, *COL5A1*, *COL12A1*, *ACAN* (ENSG00000157766), *BGN* (ENSG00000182492), *DCN* (ENSG00000011465), *FBN2*, *VEGFA*, *KDR* (ENSG00000128052) and *TGFB1* inferred functional partners have previously been associated with ligament rupture risk [24, 25, 41–52] and of these, *COL1A1*, *COL5A1*, *COL12A1*, *FBN2*, *VEGFA*, and *TGFB1* were also noted with predictive pathogenic SNPs in Family A and B (**Fig 4**).

Differentiation was observed in the distribution of minor allele frequency at rare and common variants, between the family cohort and the rest of 20 worldwide ethnic groups. Possibly, due to (1) genetic drift and population bottleneck following the recent South African apartheid, where interracial marriage was prevented; and (2) family history of ligament and other musculoskeletal soft tissue injuries that might shape the genetic makeup of the two South African families studied. Furthermore, the identification of several genes previously associated with ACL rupture, with a higher proportion of pathogenic polymorphisms (**Fig 5A**) and gene-specific in SNP frequency (**Fig 5B**), justifies and indicates that the actionability of these ACL rupture-associated genes may have differing effects on worldwide ethnic groups, supporting the beneficial use of personalised medicine, and enabling a recommendation for ACL-specific clinical actionable genes list. To our knowledge, this analysis is the first to explore such an approach in ACL research.

The study presented has provided novel insights into the genetic architecture of ACL rupture among the investigated family cohort. However, it was limited by the modest sample size of the ACL-family cohort, as larger sample sizes would most likely yield more findings. Furthermore, due to the complexity and multifactorial nature of ACL injury susceptibility, the number of affected families available for sequencing is limited. Moreover, the study was performed on isolated ACL family cases, without the possibility of variant segregation studies within the family, or in trio. Additionally, the study was limited by the availability of reliable pathogenicity prediction algorithms for intronic and intergenic variants. Therefore, it is suggested that coding variants are prioritized to facilitate this process.

Overall, the findings support the need for intensive familial studies in multiple African versus European descent populations, to unravel the novel genes and those variants that are relevant in clinical practice for diverse populations of differing genetic background. Furthermore, future work should investigate the association of these prioritised genes (**Table 1, S2 and S5 Tables**), and their potential clinical heterogeneity variants within ACL rupture risk, by utilising large case-control cohorts from the Going consortium; an established consortium to investigate the genetic susceptibility underpinning ACL rupture between different collaborating centres in the Southern and the Northern hemispheres [53]. Finally, several pathways and gene-gene interactions have been highlighted from the bioinformatic analyses and the next steps would be to explore the context of these genetic risk profiles towards a functional understanding of mechanisms underpinning risk. This can be achieved by using as an example cell culture models previously described [54, 55]. Depending on the family of proteins being investigated (structural or ECM regulators) one could evaluate differences in the (i) mechanical properties, (ii) rates of proliferation and/or (iii) migration of the derived confluent cells from the various risk profiles.”

## Conclusion

In summary, the aim to identify novel and/or previously implicated biological genomic signatures with susceptibility to ACL rupture was achieved using a WGS approach in two South African twin families, with a history of ACL rupture. From the data, a catalogue of candidate *in silico* mutations and modifier genes that clustered in pathophysiological pathways important in ACL injury, and with implications for therapeutic intervention were identified. Three candidates were implicated with *in silico* mutations clustering in potassium and ion-channel gene-families, which not only play a role in angiogenesis, but their dysfunction is known to be involved in the development of chronic painful conditions and represent key therapeutic targets. This research fills an important gap in knowledge by using a WGS approach focusing on potential deleterious coding variants, important in two unrelated families with a historical record of ACL rupture. Therefore, making significant contributions to the present knowledge of the natural history, and clinical heterogeneity of ACL rupture, with the potential for informing the design of new therapeutics.

## Materials and methods

### Ethics statement

The study recruited two South African families of Dutch/Irish (Family A) and English/Scottish (Family B) descent. All participants completed questionnaires regarding personal details, medical and sporting history, and family history of ligament and other musculoskeletal soft tissue injuries. Written informed consent was obtained from all the participants according to the declaration of Helsinki, and the study was approved by the Research Ethics Committee of the Faculty of Health Sciences within the University of Cape Town, South Africa (HREC 823/2017). All reported ACL ruptures were confirmed by either magnetic resonance imaging or surgery.

### Whole genome sequencing

Genomic DNA was isolated from venous blood according to a previously described standard protocol [56] with slight modifications [57] and prepared according to the requirements of the WGS service provider (BGI Genomics, Hong Kong). All samples passed quality control measures stipulated by BGI Genomics. In summary, for each sample the isolated DNA was fragmented, and the fragments selected by Agencourt AMPure XP-Medium kit to an average size of 200-400bp. Fragments were end repaired and 3’ adenylated and adaptor sequences ligated to the 3’ adenylated fragments. Fragments were then amplified by PCR and underwent a purification step followed by heat denaturation and circularized. Single stranded circular (ssCir) DNA formatted to a library were qualified by QC measures and sequenced using the BGISEQ-500 at 30X coverage. ssCir DNA molecule nanoballs were loaded into a patterned nanoarray using high density DNA nanochip technology, and pair end 100bp reads obtained by combinatorial Probe-Anchor Synthesis (cPAS).

### Variant calling

The Burrows-Wheeler Alignment tool [58, 59] was utilised to reconstruct reads by alignment against the complete human reference genome build hg38. Post alignment was performed using the Picard tool kit [60]. This process consisted of sorting, marking duplication reads, and the BAM files were sorted by coordinates, indexed, and read groups. Additionally, read pair information was recalculated to observe any changes by leveraging Picard FixMate Information. Bcftools [61, 62] was applied to create a clean version of the BAM files. **S1 Fig** shows the overall quality control of the bam files. Current variant calling approaches have differing advantages [63–65] and may produce differing variant calls. Here we considered an ensemble approach implemented in VariantMetaCaller [66] that may find a call consensus in detecting SNPs and short indels. The information generated from two independent variant caller pipelines: (1) An incremental joint variant discovery implemented in GATK 3.0 HaplotypeCaller [60] which calls samples independently to produce gVCF files and leverages the information from the independent gVCF file to produce a call-set at the genotyping step; (2) bcftools via mpileup [61, 62, 67] was performed to produce an additional genotyped call-set. The best practice specific to each caller was adopted [68]. Before applying the ensemble approach from the resulting variant sets from the callers above, each resulting VCF file was filtered using the GATK 3.0 tool Variant Filtration. The final call-set was produced from VariantMetaCaller [66] a support vector machines approach that combines the hard-filtered VCF files obtained from these 2 variant callers.

### Annotation, in silico prediction of mutation and prioritization

ANNOVAR [69] was used to perform gene-based annotation to detect whether the SNPs detected resulted in protein coding changes and to produce a list of the affected amino acids. Population frequency and pathogenicity for each variant was obtained from 1000 Genomes exome (1000 Genomes Project Consortium, 2012), Exome Aggregation Consortium (ExAC) [70] targeted exon datasets and COSMIC [71]. Gene functions were obtained from RefGene [72] and different functional predictions were obtained from ANNOVAR’s library, which contains up to 21 different functional scores including SIFT [73, 74] LRT [75] MutationTaster [76] MutationAssessor [77, 78] FATHMM [79] fathmm-MKL [79] RadialSVM [80] LR [80] PROVEAN [80] MetaSVM [80] MetaLR [80] CADD [81] GERP++ [82] DANN, M-CAP, Eigen, GenoCanyon, Polyphen2 HVAR [83] Polyphen2 HDIV [84] PhyloP [83] and SiPhy (Garber et al., 2009). Additionally, conservative, and segmental duplication sites, dbSNP code and clinical relevance reported in dbSNP [85] were also included. From the resulting functional annotated dataset, we first filtered variants for rarity, exonic variants, non-synonymous, stop codons, predicted functional significance and deleteriousness [73, 74]. The resulting functional annotated data set was independently filtered for predicted functional status (of which each predicted functional status is of "deleterious" (D), "probably damaging" (D), "disease_causing_automatic" (A) or "disease_causing" (D). [86–88] from these 21 in silico prediction mutation tools. A casting vote approach was utilized to retain only a variant if it had at least 17 predicted functional status “D” or “A”out of 21. The retained variants were further filtered for rarity, exonic variants, nonsynonymous mutations, yielding a final candidate list of predicted mutant and genetics modifier variants.

### Phased and haplotypes inference

Additional VCF files were accessed from the 1000 Genomes Project Consortium, 2015 and the African Genome Variation Project (AGVP) which recently characterized the admixture across 20 ethno-linguistic groups (**S6 Table**) from sub-Saharan Africa [89]. PLINK was used to carry out quality control on the VCF files, and in total 2,504 BAM files from 1000 Genomes Project and 2,428 from AGVP were retained. Based on the initial sample description (population or country labels, we used the population ethno-linguistic information [90, 91] to categorize the obtained data per ethnic group as described in. We merged our family datasets with the available data from 20 worldwide ethnic groups regardless of depth of coverage.

To increase the accuracy, the resulting VCF file contained 4,932 samples of 20 ethnic groups, were used to further conduct quality control in removing all structured, indel, multi-allelic variants and those with low minor allele frequency (MAF < 0.05) prior to phasing. We first phased and inferred the haplotypes using Eagle from the resulting curated data [92]. We further compared sites discordance between these haplotype panels and independently with their original VCF file prior phasing. The only site with phase switch-errors showed discrepancies in MAF and was removed.

### Principal component analysis

Principal components analysis (PCA) is now routinely used to detect and quantify the genetic structure of populations. We performed LD pruning on our merged family dataset using PLINK to remove correlated (r^2^ >0.15) SNPs in a 1,000-SNP window, advancing by 10 SNPs at a time. The pruned dataset contained 9,487,525 SNPs and 9 individuals with a genotype call rate of 99.9%. We accessed VCF files of 2,504 samples from 1000 Genomes Project Consortium, 2015 and 2,428 samples from the African Genome Variation Project (AGVP) which has recently characterized the admixture across 18 ethno-linguistic groups from sub-Saharan Africa [89]. We conducted a quality control check on these VCF files using plink and vcftools. Based on initial sample description (population or country labels), we used the population ethno-linguistic information to categorize the obtained data per ethnic group. We merged our family datasets and these ethnic groups’ data set, yield to 45,096 SNPs, on 4,941 samples in 20 ethnic groups. We used smartpca to perform PCA on the pruned family dataset and the merged data set of 20 worldwide ethnic groups. We use GENESIS (v0.2.6b) (http://www.bioinf.wits.ac.za/software/genesis) for plot visualizations.

### Percentage pathogenic SNPS within previously implicated genes

To evaluate the distribution of pathogenic variants across worldwide ethnics and our family dataset within genes known to be associated with risk of ligament and tendon injuries (**S3 Table**), we leveraged the dbSNP database to extract SNPs associated with these genes. The obtained SNPs were extracted from the merged phased data containing 4,941 samples from 20 ethnic groups and our family’s data set. The resulting data were split into each ethnic group and annotation using ANNOVAR were performed as described above. The fraction of pathogenic within a gene was approximated as the count of reported pathogenic SNPs from ANNOVAR divided by the total count of associated SNPs to the gene from dbSNPs.

### Distribution of minor allele frequency and gene-specific SNP frequencies

To examine the extent of how common SNPs are distributed across these 20 ethnic groups on genes known to be associated with our phenotype of interest, we investigated the distribution of the minor allele frequency. To this end, the proportion of minor alleles were categorized into 6 bins (0–0.05, >0.05–0.1, >0.1–0.2, >0.2–0.3, >0.3–0.4, >0.4–0.5) with respect to each ethnic group with a disease. The minor allele frequency (MAF) per SNP for each category was computed using Plink software. Furthermore, the fraction of gene-specific SNP frequency for each gene was computed, assuming SNPs upstream and downstream within the associated gene region as annotated from dbSNP database. Minor allele frequency per SNP was aggregated at the gene level as from our previous works [93].

### Identity by descent and functional genomics

Haplotypes are identical by descent if they are identical and inherited from a common ancestor. Tracts of identity by descent (IBD) are broken up by recombination during meiosis, so expected length of IBD depends on the number of generations since the common ancestor for the locus. If the common ancestor lived a great many generations ago, the individuals share very short tracts of genetic material. Accurate estimation of genomic IBD sharing depends not only on detection of IBD tracts but also on accurate estimation of the ends of those tracts and modelling linkage disequilibrium. Here, we used the two-family data separately, to investigate the overall genomic identity-by-descent (IBD) sharing between pairs of individuals within each family and thus across families. The segments of IBD were obtained using the Refined IBD algorithm [94]. The genomic IBD segments within families were evaluated and the shared segments between the two families compared. The potential functional roles of the genes localised to these shared IBDs and additional selected genes were explored using network and enrichment analysis, to gain insight into potential disease compromised networks. To explore functional partners, GeneCards [95] premium analytical tool; *GenesLikeMe*, was utilised, where a weighting of 3 (out of 5) was set for each attribute (sequence paralogs, domains, super pathways, expression patterns, phenotypes, compounds, disorders and gene ontologies). From the results, the top 100 inferred functional partners were further enriched for common sub-networks, pathways, biological processes, molecular functions, and association to potential human phenotypes using Enrichr software [96]. Finally, functional partners were summarised and grouped by protein function.

### Network and enrichment analysis

From the retained final candidate list of predicted mutant and genetics modifier variants, how each set of the variant genes are layered and interact within a biological network was examined. This was carried out using a comprehensive human Protein-Protein Interaction (PPI) network to identify sub-networks of interactive mutant and genetic modifier variant genes [19, 97, 98]. Furthermore, Enrichr software [96] was again utilised to determine how these genes interact in sub-networks, their pathways, biological processes, molecular functions, and association to potential human phenotypes. The most significant pathway enriched for genes in the network were selected from KEGG [99] Panther [100] Biocarta [101] and Reactome [102] and gene ontologies from the Gene Ontology Consortium (Reference Genome Group of the Gene Ontology Consortium, 2009) were defined for cellular component, biological process, and molecular function.

### 3D protein structure prediction for functional characterization of novel variants

Molecular dynamic (MD) simulations were conducted to assess the effect of novel variants on protein function for *KCNJ12* and *CATSPER*. The amino acid sequences were obtained from UniProt for *KCNJ12* (https://www.uniprot.org/uniprot/Q14500) and *CATSPER2* (https://www.ebi.ac.uk/ena/browser/api/fasta/AF411817.1?lineLimit=1000). The tertiary structure of *KCNJ12* and *CATSPER2* genes were generated using I-tasser homology webserver [103]. All MD simulations were conducted with the GROMACS package, version 5.6. [104–107] using Amber (AMBER99SB-ILDN) force field [108]. The system was solvated in dodecahedron box of tip4p water. The temperature and pressure were maintained at 300 K using the Parrinello-Donadio-Bussi V-rescale thermostat [109] and a pressure of 1bar using the Berendsen barostat [110]. The short-range non-bonded interactions were modelled using Lennard Jones potentials. The long-range electrostatic interactions were calculated using the particle mesh Ewald (PME) algorithm [111, 112]. The LINCS algorithm was used to constrain hydrogen bond lengths [113] and the velocities assigned according to the Maxwell-Boltzman distribution at 300 K. The equilibration of the structure NVT (constant Number of particles, Volume, and Temperature) and NPT (constant Number of particles, Pressure, and Temperature) for 10 ns each. The MD production simulations were run for 50 ns for each structure.

## Supporting information

S1 Fig Overall quality control data of the bam files. (TIF)

S2 Fig 3D structure superposition of *CATSPER2* protein site comparing the wild type (green) and mutant (cyan) forms. (TIF)

S3 Fig Minor allele frequencies at rare and common variants between Family A and Family B, and the rest of 20 worldwide ethnic groups. (TIF)

S4 Fig Linkage disequilibrium around *LINC01250* gene. (TIF)

S1 Table History of other musculoskeletal soft tissue injuries in Family A and Family B. (DOCX)

S2 Table Candidate list of genes with predicted mutations in Family A and Family B. Genes in bold depict predicted pathogenic mutations shared in Family A and B.
(DOCX)

S3 Table Genes previously associated with ligament and tendon injury. (DOCX)

S4 Table The table displays the top significant pathways, GO biological process, molecular function and human phenotypes associated with the genes previously associated with tendon and ligament injury, and the candidate list of predicted pathogenic genes and their interacting genes for Family A and Family B combined and independently. (DOCX)

S5 Table Inferred functional partners and enriched pathways for genes of interest in Family A and B. Genes in bold script: highlighted as previously associated with musculoskeletal injury susceptibility.
(DOCX)

S6 Table Data obtained from 1000 Genomes Project (1KGP) (Consortium et al., 2012) and the African Genome Variation Project (AGVP) (Gurdasani et al., 2015) used for analysis. (DOCX)

S1 File (PDF)

## References

[1] Wang Y, Wang J. Modelling and prediction of global non-communicable diseases. BMC public health. 2020;20(1):822. doi: 10.1186/s12889-020-08890-4 32487173 PMC7268487

[2] Lee IM, Shiroma EJ, Lobelo F, Puska P, Blair SN, Katzmarzyk PT, et al. Effect of physical inactivity on major non-communicable diseases worldwide: an analysis of burden of disease and life expectancy. Lancet 2012;380(9838):219–29. doi: 10.1016/S0140-6736(12)61031-9 22818936 PMC3645500

[3] Safiri S, Kolahi A-A, Cross M, Hill C, Smith E, Carson-Chahhoud K, et al. Prevalence, Deaths, and Disability-Adjusted Life Years Due to Musculoskeletal Disorders for 195 Countries and Territories 1990–2017. Arthritis Rheumatol. 2021;73:702–14. doi: 10.1002/art.41571 33150702

[4] Gianotti SM, Marshall SW, Hume PA. Incidence of anterior cruciate ligament injury and other knee ligament injuries: a national population-based study. J Sci Med Sport. 2009;12(6):622–27. doi: 10.1016/j.jsams.2008.07.005 18835221

[5] Ageberg E. Consequences of a ligament injury on neuromuscular function and relevance to rehabilitation—Using the anterior cruciate ligament-injured knee as model. J Electromyogr Kinesiol. 2002;12(3):205–12. doi: 10.1016/s1050-6411(02)00022-6 12086815

[6] Pfeifer CE, Beattie PF, Sacko RS, Hand A. Risk factors associated with non-contact anterior cruciate ligament injury: a systematic review. Int J Sports Phys Ther. 2018;13(4):575–87. 30140551 PMC6088120

[7] Griffin LY, Albohm MJ, Arendt EA. Understanding and preventing noncontact anterior cruciate ligament injuries: a review of the Hunt Valley II Meeting, Januaury 2005. Am J Sports Med. 2006;34(9):1512–32. 16905673 10.1177/0363546506286866

[8] Kaynak M, Nijman F, van Meurs J, Reijman M, Meuffels DE. Genetic Variants and Anterior Cruciate Ligament Rupture: A Systematic Review. Sports Med. 2017;47(8):1637–50. doi: 10.1007/s40279-017-0678-2 28102489 PMC5507974

[9] Rahim M, Gibbon A, Collins M, September AV. Genetics of musculoskeletal soft tissue injuries: current status, challenges, and future directions. In: Barh D, Ahmetov I, editors. Sports, Exercise, and Nutritional Genomics. Cambridge, MA: Academic Press; 2019. p. 317‐39.

[10] Magnusson K, Turkiewicz A, Hughes V, Frobell R, Englund M. High genetic contribution to anterior cruciate ligament rupture: Heritability ~69%. Br J Sports Med. 2020;55:385–89. doi: 10.1136/bjsports-2020-102392 33288618

[11] Baird AEG, Carter SD, Innes JF, Ollier W, Short A. Genome-wide association study identifies genomic regions of association for cruciate ligament rupture in Newfoundland dogs. Anim Genet. 2014;45(4):542–49. doi: 10.1111/age.12162 24835129

[12] Baker L, Kirkpatrick B, Rosa G, Gianola D, Valente B, Sumner J, et al. Genome-wide association analysis in dogs implicates 99 loci as risk variants for anterior cruciate ligament rupture. PLoS One. 2017;12(4):1–19. doi: 10.1371/journal.pone.0173810 28379989 PMC5381864

[13] Baker LA, Rosa GJM, Hao Z, Piazza A, Hoffman C, Binversie EE, et al. Multivariate genome-wide association analysis identifies novel and relevant variants associated with anterior cruciate ligament rupture risk in the dog model. BMC Genet. 2018;19(1):39. doi: 10.1186/s12863-018-0626-7 29940858 PMC6019516

[14] Kim S, Roos T, Roos A, Kleimeyer J, Ahmed M, Goodlin G, et al. Genome-wide association screens for Achilles tendon and ACL tears and tendinopathy. PLoS One. 2017;12(3):1–16. doi: 10.1371/journal.pone.0170422 28358823 PMC5373512

[15] Kim SK, Nguyen C, Avins AL, Abrams GD. Three genes associated with anterior and posterior cruciate ligament injury. Bone Jt Open. 2021;2(6):414–21. 34169730 10.1302/2633-1462.26.BJO-2021-0040.R1 PMC8244791

[16] Caso E, Maestro A, Sabiers CC, Godino M, Caracuel Z, Pons J, et al. Whole-exome sequencing analysis in twin sibling males with an anterior cruciate ligament rupture. Injury. 2016;47:S41–S50. doi: 10.1016/S0020-1383(16)30605-2 27692106

[17] Hunt J. Dutch South Africa: Early Settlers at the Cape 1652–1708. Philadelphia: University of Pennsylvania Press; 2005.

[18] Bouaoun L, Sonkin D, Ardin M, Hollstein M, Byrnes G, Zavadil J, et al. TP53 Variations in Human Cancers: New Lessons from the IARC TP53 Database and Genomics Data. Hum Mutat. 2016;37(9):865–76. doi: 10.1002/humu.23035 27328919

[19] Warde-Farley D, Donaldson SL, Comes O, Zuberi K, Badrawi R, Chao P, et al. The GeneMANIA prediction server: biological network integration for gene prioritization and predicting gene function. Nucleic acids research. 2010;38(Web Server issue), W214–W220. doi: 10.1093/nar/gkq537 20576703 PMC2896186

[20] Birk DE, Fitch JM, Babiarz JP, Doane KJ, Linsenmayer TF. Collagen fibrillogenesis in vitro: interaction of types I and V collagen regulates fibril diameter. J Cell Sci. 1990;95:649–57. doi: 10.1242/jcs.95.4.649 2384532

[21] Young BB, Zhang G, Koch M, Birk DE. The roles of types XII and XIV collagen in fibrillogenesis and matrix assembly in the developing cornea. J Cell Biochem. 2002;87(2):208–20. doi: 10.1002/jcb.10290 12244573

[22] Frank CB. Ligament structure, physiology and function. J Musculoskelet Neuronal Interact. 2004;4(2):199–201. 15615126

[23] Ficek K, Stepien-Slodkowska M, Kaczmarczyk M, Maciejewska-Karlowska A, Sawczuk M, Cholewinski J, et al. Does the A9285G Polymorphism in Collagen Type XII α1 Gene Associate with the Risk of Anterior Cruciate Ligament Ruptures? Balkan J Med Genet. 2014;17(1):41–6. doi: 10.2478/bjmg-2014-0022 25741214 PMC4347476

[24] O’Connell K, Knight H, Ficek K, Leonska-Duniec A, Maciejewska-Karlowska A, Sawczuk M, et al. Interactions between collagen gene variants and risk of anterior cruciate ligament rupture. Eur J Sport Sci. 2015;15(4):341–50. doi: 10.1080/17461391.2014.936324 25073002

[25] Posthumus M, September AV, Cuinneagain DO, Van Der Merwe W, Schwellnus MP, Collins M. The association between the COL12A1 gene and anterior cruciate ligament ruptures. Br J Sports Med. 2010;44(16):1160–65. doi: 10.1136/bjsm.2009.060756 19443461

[26] Sivertsen EA, Haug K, Kristianslund EK, Trøseid AS, Parkkari J, Lehtimäki T, et al. No Association Between Risk of Anterior Cruciate Ligament Rupture and Selected Candidate Collagen Gene Variants in Female Elite Athletes From High-Risk Team Sports. Am J Sports Med. 2019;47(1):52–8. doi: 10.1177/0363546518808467 30485117

[27] Zhao D, Zhang Q, Lu Q, Hong C, Luo T, Duan Q, et al. Correlations Between the Genetic Variations in the COL1A1, COL5A1, COL12A1, and β-fibrinogen Genes and Anterior Cruciate Ligament Injury in Chinese Patientsa. J Athl Train. 2020;55(5):515–21. doi: 10.4085/1062-6050-335-18 32239963 PMC7249278

[28] Kim J. Channelopathies. Korean J Pediatr. 2014;57(1):1–18. doi: 10.3345/kjp.2014.57.1.1 24578711 PMC3935107

[29] Schmidt AP, Schmidt SRG. Behavior of ion channels controlled by electric potential difference and of Toll-type receptors in neuropathic pain pathophysiology. Revista Dor. 2016;177:13–45.

[30] Waxman SG, Merkies ISJ, Gerrits MM, Dib-Hajj SD, Lauria G, Cox JJ, et al. Sodium channel genes in pain-related disorders: phenotype–genotype associations and recommendations for clinical use. Lancet Neurol. 2014;13(11):1152–60. doi: 10.1016/S1474-4422(14)70150-4 25316021

[31] Biasiotta A, D’Arcangelo D, Passarelli F, Nicodemi EM, Facchiano A. Ion channels expression and function are strongly modified in solid tumors and vascular malformations. J Transl Med. 2016;14(1):1–15. doi: 10.1186/s12967-016-1038-y 27716384 PMC5050926

[32] Franklin BM, Voss SR, Osborn JL. Ion channel signaling influences cellular proliferation and phagocyte activity during axolotl tail regeneration. Mech Dev. 2017;146:42–54. doi: 10.1016/j.mod.2017.06.001 28603004 PMC6386162

[33] Nilius B, Droogmans G. Ion channels and their functional role in vascular endothelium. Physiol Rev. 2001;81(4):1415–59. doi: 10.1152/physrev.2001.81.4.1415 11581493

[34] Hibino H, Inanobe A, Furutani K, Murakami S, Findlay I, Kurachi Y. Inwardly rectifying potassium channels: their structure, function, and physiological roles. Physiol Rev. 2010;90(1):291–366. doi: 10.1152/physrev.00021.2009 20086079

[35] Yoshikawa T, Tohyama H, Katsura T, Kondo E, Kotani Y, Matsumoto H, et al. Effects of local administration of vascular endothelial growth factor on mechanical characteristics of the semitendinosus tendon graft after anterior cruciate ligament reconstruction in sheep. Am J Sports Med. 2006;34(12):1918–25. doi: 10.1177/0363546506294469 17092923

[36] Du X, Gamper N. Potassium channels in peripheral pain pathways: expression, function and therapeutic potential. Curr Neuropharmacol. 2013;11(6):621–40. doi: 10.2174/1570159X113119990042 24396338 PMC3849788

[37] Karthaus M, Schiel X, Ruhlmann CH, Celio L. Neurokinin-1 receptor antagonists: review of their role for the prevention of chemotherapy-induced nausea and vomiting in adults. Expert review of clinical pharmacology. Expert Rev Clin Pharmacol. 2019;12(7):661–80. 31194593 10.1080/17512433.2019.1621162

[38] Vega L, Mateyak M, Zakian V. Getting to the end: telomerase access in yeast and humans. Nat Rev Mol Cell Biol. 2003;4(12):948–59. doi: 10.1038/nrm1256 14685173

[39] Ransohoff JD, Wei Y, Khavari PA. The functions and unique features of long intergenic non-coding RNA. Nat Rev Mol Cell Biol. 2018;19(3):143–57. doi: 10.1038/nrm.2017.104 29138516 PMC5889127

[40] Luan A, Hu MS, Leavitt T, Brett EA, Wang KC, Longaker MT, et al. Noncoding RNAs in Wound Healing: A New and Vast Frontier. Adv Wound Care. 2018;7(1):19–27. doi: 10.1089/wound.2017.0765 29344431 PMC5770091

[41] Ficek K, Cieszczyk P, Kaczmarczyk M, Maciejewska-Karłowska A, Sawczuk M, Cholewinski J, et al. Gene variants within the COL1A1 gene are associated with reduced anterior cruciate ligament injury in professional soccer players. J Sci Med Sport. 2013;16(5):396–400. doi: 10.1016/j.jsams.2012.10.004 23168334

[42] Gibbon A, Raleigh SM, Ribbans WJ, Posthumus M, Collins M, September AV. Functional COL1A1 variants are associated with the risk of acute musculoskeletal soft tissue injuries. J Orthop Res. 2020;38(10):2290–98. doi: 10.1002/jor.24621 32017203

[43] Stępień-Słodkowska M, Ficek K, Kaczmarczyk M, Maciejewska A, Sawczuk M, Eider J, et al. Influence of biological factors on injuries occurrence in the Polish population. Ann Agric Environ Med. 2016;23(2):315–8. doi: 10.5604/12321966.1203897 27294639

[44] Lulińska-Kuklik E, Rahim M, Domańska-Senderowska D, Ficek K, Michałowska-Sawczyn M, Moska W, et al. Interactions between COL5A1 Gene and Risk of the Anterior Cruciate Ligament Rupture. J Hum Kinet. 2018;62:65–71. doi: 10.1515/hukin-2017-0177 29922378 PMC6006531

[45] Posthumus M, September AV, Schwellnus MP, Collins M. Investigation of the Sp1-binding site polymorphism within the COL1A1 gene in participants with Achilles tendon injuries and controls. J Sci Med Sport. 2009;12(1):184–9. doi: 10.1016/j.jsams.2007.12.006 18353721

[46] Wang C, Li H, Chen K, Wu B, Liu H. Association of polymorphisms rs1800012 in COL1A1 with sports-related tendon and ligament injuries: a meta-analysis. Oncotarget. 2017;8(16):27627–34. doi: 10.18632/oncotarget.15271 28206959 PMC5432363

[47] Posthumus M, September AV, O’Cuinneagain D, Van der Merwe W, Schwellnus MP, Collins M. The COL5A1 Gene Is Associated With Increased Risk of Anterior Cruciate Ligament Ruptures in Female Participants. Am J Sports Med. 2009;37(11):2234–40. doi: 10.1177/0363546509338266 19654427

[48] El Khoury L, Posthumus M, Collins M, van der Merwe W, Handley CJ, Cook J, et al. ELN and FBN2 gene variants as risk factors for two sports-related musculoskeletal injuries. Int J Sports Med. 2015;36(4):333–7. doi: 10.1055/s-0034-1390492 25429546

[49] Rahim M, Gibbon A, Hobbs H, van der Merwe W, Posthumus M, Collins M, et al. The association of genes involved in the angiogenesis-associated signaling pathway with risk of anterior cruciate ligament rupture. J Orthop Res. 2014;32(12):1612–8. doi: 10.1002/jor.22705 25111568

[50] Laguette MN, Barrow K, Firfirey F, Dlamini S, Saunders CJ, Dandara C, et al. Exploring new genetic variants within COL5A1 intron 4‐exon 5 region and TGF‐β family with risk of anterior cruciate ligament ruptures. J Orthop Res. 2020;38(8):1856–65. doi: 10.1002/jor.24585 31922278

[51] Cięszczyk P, Willard K, Gronek P, Zmijewski P, Trybek G, Gronek J, et al. Are genes encoding proteoglycans really associated with the risk of anterior cruciate ligament rupture? Biol Sport. 2017;34(2):97–103. doi: 10.5114/biolsport.2017.64582 28566802 PMC5424448

[52] Mannion S, Mtintsilana A, Posthumus M, van der Merwe W, Hobbs H, Collins M, et al. Genes encoding proteoglycans are associated with the risk of anterior cruciate ligament ruptures. Br J Sport Med. 2014;48(22):1640–6. doi: 10.1136/bjsports-2013-093201 24552666

[53] Pitsiladis YP, Tanaka M, Eynon N, Bouchard C, North KN, Williams AG, et al. Athlome Project Consortium: a concerted effort to discover genomic and other “omic” markers of athletic performance. Physiol Genomics. 2016;48(3):183–90. doi: 10.1152/physiolgenomics.00105.2015 26715623 PMC4773890

[54] Suijkerbuijk MAM, Ponzetti M, Rahim M, Posthumus M, Häger CK, Stattin E, et al. Functional polymorphisms within the inflammatory pathway regulate expression of extracellular matrix components in a genetic risk dependent model for anterior cruciate ligament injuries. J Sci Med Sport. 2019;22(11):1219–25. doi: 10.1016/j.jsams.2019.07.012 31395468

[55] Willard K, Laguette MN, Alves de Souza Rios L, D’Alton C, Nel M, Prince S, et al. Altered expression of proteoglycan, collagen and growth factor genesin a TGF-β1 stimulated genetic risk model for musculoskeletal softtissue injuries. J Sci Med Sport. 2020;23(8):695–700. doi: 10.1016/j.jsams.2020.02.007 32061523

[56] Lahiri DK, Nurnberger JJI. A rapid non-enzymatic method for the preparation of HMW DNA from blood for RFLP studies. Nucleic Acids Res. 1991;19(19):5444. doi: 10.1093/nar/19.19.5444 1681511 PMC328920

[57] Mokone GG, Gajjar M, September AV, Schwellnus MP, Greenberg J, Noakes TD, et al. The guanine-thymine dinucleotide repeat polymorphism within the tenascin-C gene is associated with achilles tendon injuries. Am J Sports Med. 2005;33(7):1016–21. doi: 10.1177/0363546504271986 15983124

[58] Li H, Ruan J, Durbin R. Mapping short DNA sequencing reads and calling variants using mapping quality scores. Genome Res. 2008;18(11):1851–8. doi: 10.1101/gr.078212.108 18714091 PMC2577856

[59] Li H, Durbin R. Fast and accurate short read alignment with Burrows-Wheeler transform. Bioinformatics. 2009;25(14):1754–60. doi: 10.1093/bioinformatics/btp324 19451168 PMC2705234

[60] McKenna A, Hanna M, Banks E, Sivachenko A, Cibulskis K, Kernytsky A, et al. The Genome Analysis Toolkit: a MapReduce framework for analyzing next-generation DNA sequencing data. Genome Res. 2010;20(9):1297–303. doi: 10.1101/gr.107524.110 20644199 PMC2928508

[61] Narasimhan V, Danecek P, Scally A, Xue Y, Tyler-Smith C, Durbin R. BCFtools/RoH: a hidden Markov model approach for detecting autozygosity from next-generation sequencing data. Bioinformatics. 2016;32(11):1749–51. doi: 10.1093/bioinformatics/btw044 26826718 PMC4892413

[62] Danecek P, McCarthy SA. BCFtools/csq: haplotype-aware variant consequences. Bioinformatics. 2017;33(13):2037–39. doi: 10.1093/bioinformatics/btx100 28205675 PMC5870570

[63] DePristo MA, Banks E, Poplin R., Garimella KV, Maguire JR, Hartl C, Philippakis A. A., del Angel G, et al. A framework for variation discovery and genotyping using next-generation DNA sequencing data. Nat Genet. 2011;43(5):491–8. doi: 10.1038/ng.806 21478889 PMC3083463

[64] Liu Q, Guo Y, Li J, Long J, Zhang B, Shyr Y. Steps to ensure accuracy in genotype and SNP calling from Illumina sequencing data. BMC genomics. 2012;13 Suppl 8(Suppl 8)(S8). doi: 10.1186/1471-2164-13-S8-S8 23281772 PMC3535703

[65] Liu X, Han S, Wang Z, Gelernter J, Yang BZ. Variant callers for next-generation sequencing data: a comparison study. PloS One. 2013;8(9):e75619. doi: 10.1371/journal.pone.0075619 24086590 PMC3785481

[66] Gézsi A, Bolgár B, Marx P, Sarkozy P, Szalai C, Antal P. VariantMetaCaller: automated fusion of variant calling pipelines for quantitative, precision-based filtering. BMC genomics. 2015;16:875. doi: 10.1186/s12864-015-2050-y 26510841 PMC4625715

[67] Garrison E, Marth G. Haplotype-based variant detection from short-read sequencing. arXiv preprint arXiv. 2012;1207:3907

[68] Cornish A, Guda C. A Comparison of Variant Calling Pipelines Using Genome in a Bottle as a Reference. Biomed Res Int. 2015:456479. doi: 10.1155/2015/456479 26539496 PMC4619817

[69] Wang K, Li M, Hakonarson H. ANNOVAR: functional annotation of genetic variants from high-throughput sequencing data. Nucleic Acids Res. 2010;38(16):e164. doi: 10.1093/nar/gkq603 20601685 PMC2938201

[70] Karczewski KJ, Weisburd B, Thomas B, Solomonson M, Ruderfer DM, Kavanagh D, et al. The ExAC browser: displaying reference data information from over 60 000 exomes. Nucleic Acids Res. 2017;45(D1):D840–D5. doi: 10.1093/nar/gkw971 27899611 PMC5210650

[71] Forbes SA, Beare D, Gunasekaran P, Leung K, Bindal N, Boutselakis H, et al. COSMIC: Exploring the world’s knowledge of somatic mutations in human cancer. Nucleic Acids Res. 2015;43:D805–D11. doi: 10.1093/nar/gku1075 25355519 PMC4383913

[72] O’Leary NA, Wright MW, Brister JR, Ciufo S, Haddad D, McVeigh R, et al. Reference sequence (RefSeq) database at NCBI: Current status, taxonomic expansion, and functional annotation. Nucleic Acids Res. 2016;44:D733–D45. doi: 10.1093/nar/gkv1189 26553804 PMC4702849

[73] Ng PC, Henikoff S. SIFT: Predicting amino acid changes that affect protein function. Nucleic Acids Res. 2003;31(13):3812–4. doi: 10.1093/nar/gkg509 12824425 PMC168916

[74] Ng PC, Henikoff S. Predicting the effects of amino acid substitutions on protein function. Annu Rev Genomics Hum Genet. 2006;7:61–80. doi: 10.1146/annurev.genom.7.080505.115630 16824020

[75] Fujita A, Kojima K, Patriota AG, Sato JR, Severino P, Miyano S. A fast and robust statistical test based on likelihood ratio with Bartlett correction to identify Granger causality between gene sets. Bioinformatics. 2010;26(18):2349–51. doi: 10.1093/bioinformatics/btq427 20660295

[76] Schwarz JM, Cooper DN, Schuelke M, Seelow D. MutationTaster2: mutation prediction for the deep-sequencing age. Nat Methods. 2014;11(4):361–2. doi: 10.1038/nmeth.2890 24681721

[77] Reva B, Antipin Y, Sander C. Determinants of protein function revealed by combinatorial entropy optimization. Genome Biol. 2007;8(11):R232. doi: 10.1186/gb-2007-8-11-r232 17976239 PMC2258190

[78] Reva B, Antipin Y, Sander C. Predicting the functional impact of protein mutations: Application to cancer genomics. Nucleic Acids Res. 2011;39(17):37–43. doi: 10.1093/nar/gkr407 21727090 PMC3177186

[79] Shihab HA, Gough J, Mort M, Cooper DN, Day INM, Gaunt TR. Ranking non-synonymous single nucleotide polymorphisms based on disease concepts. Hum Genomics. 2014;8(1):11. doi: 10.1186/1479-7364-8-11 24980617 PMC4083756

[80] Dong C, Wei P, Jian X, Gibbs R, Boerwinkle E, Wang K, et al. Comparison and integration of deleteriousness prediction methods for nonsynonymous SNVs in whole exome sequencing studies. Hum Mol Genet. 2015;24(8):2125–37. doi: 10.1093/hmg/ddu733 25552646 PMC4375422

[81] Kircher M, Witten DM, Jain P, O’Roak BJ, Cooper GM, Shendure J. A general framework for estimating the relative pathogenicity of human genetic variants. Nat Genet. 2014;46(3):310–5. doi: 10.1038/ng.2892 24487276 PMC3992975

[82] Cooper GM, Stone EA, Asimenos G, NISC Comparative Sequencing Program G, E. D., Batzoglou S, Sidow A. Distribution and intensity of constraint in mammalian genomic sequence. Genome Res. 2005;15(7):901–13. 15965027 10.1101/gr.3577405 PMC1172034

[83] Garber M, Guttman M, Clamp M, Zody MC, Friedman N, Xie X. Identifying novel constrained elements by exploiting biased substitution patterns. Bioinformatics. 2009;25(12):54–62. doi: 10.1093/bioinformatics/btp190 19478016 PMC2687944

[84] Adzhubei IA, Schmidt S, Peshkin L, Ramensky VE, Gerasimova A, Bork P, et al. A method and server for predicting damaging missense mutations. Nat Methods. 2010;7(4):248–9. doi: 10.1038/nmeth0410-248 20354512 PMC2855889

[85] Sherry ST, Ward MH, Kholodov M, Baker J, Phan L, Smigielski EM, et al. dbSNP: the NCBI database of genetic variation. Nucleic Acids Res. 2001;29(1):308–11. doi: 10.1093/nar/29.1.308 11125122 PMC29783

[86] Wheeler DL, Barrett T, Benson DA, Bryant SH, Canese K, Chetvernin V, et al. Database resources of the National Center for Biotechnology Information. Nucleic Acids Res. 2008;35:D13–D21. doi: 10.1093/nar/gkm1000 18045790 PMC2238880

[87] Sifrim A, Popovic D, Tranchevent LC, Ardeshirdavani A, Sakai R, Konings P, et al. eXtasy: variant prioritization by genomic data fusion. Nat Methods. 2013;10(11):1083–4. doi: 10.1038/nmeth.2656 24076761

[88] Li MX, Kwan JS, Bao SY, Yang W, Ho SL, Song YQ, et al. Predicting mendelian disease-causing non-synonymous single nucleotide variants in exome sequencing studies. PLoS Genet. 2013;9(1):e1003143. doi: 10.1371/journal.pgen.1003143 23341771 PMC3547823

[89] Gurdasani D, Carstensen T, Tekola-Ayele F, Pagani L, Tachmazidou I, Hatzikotoulas K, et al. The African Genome Variation Project shapes medical genetics in Africa. Nature. 2015;517(7534):327–32. doi: 10.1038/nature13997 25470054 PMC4297536

[90] Gudykunst WB, Schmidt KL. Language and ethnic identity: An overview and prologue. J Lang Soc Psychol. 1987;6(3–4):157–70.

[91] Michalopoulos S. The Origins of Ethnolinguistic Diversity. Am Econ Rev. 2012;102(4):1508–39. doi: 10.1257/aer.102.4.1508 25258434 PMC4172340

[92] Loh PR, Palamara PF, Price AL. Fast and accurate long-range phasing in a UK Biobank cohort. Nature Genet. 2016;48(7):811–6. doi: 10.1038/ng.3571 27270109 PMC4925291

[93] Wonkam A, Chimusa ER, Mnika K, Pule GD, Ngo Bitoungui VJ, Mulder N, et al. Genetic modifiers of long‐term survival in sickle cell anemia. Clin Transl Med. 2020;10(4):1–15. doi: 10.1002/ctm2.152 32898326 PMC7423184

[94] Browning BL, Browning SR. Improving the accuracy and efficiency of identity-by-descent detection in population data. Genetics. 2013;194(2):459–71. doi: 10.1534/genetics.113.150029 23535385 PMC3664855

[95] Stelzer G, Rosen R, Plaschkes I, Zimmerman S, Twik M, Fishilevich S, et al. The GeneCards Suite: From Gene Data Mining to Disease Genome Sequence Analysis. Curr Protoc Bioinformatics. 2016;54(1). 10.1002/cpbi.5 27322403

[96] Kuleshov MV, Jones MR, Rouillard AD, Fernandez NF, Duan Q, Wang Z, et al. Enrichr: a comprehensive gene set enrichment analysis web server 2016 update. Nucleic Acids Res. 2016;8(44):90–7. doi: 10.1093/nar/gkw377 27141961 PMC4987924

[97] Montojo J, Zuberi K, Rodriguez H, Bader GD, Morris Q. GeneMANIA: Fast gene network construction and function prediction for Cytoscape. F1000Res. 2014;3:153. doi: 10.12688/f1000research.4572.1 25254104 PMC4168749

[98] Franz M, Rodriguez H, Lopes C, Zuberi K, Montojo J, Bader GD, et al. GeneMANIA update 2018. Nucleic Acids Res. 2018;46(W1):W60–W4. doi: 10.1093/nar/gky311 29912392 PMC6030815

[99] Kanehisa M, Goto S. KEGG: kyoto encyclopedia of genes and genomes. Nucleic Acids Res. 2000;28(1):27–30. doi: 10.1093/nar/28.1.27 10592173 PMC102409

[100] Mi H, Huang X, Muruganujan A, Tang H, Mills C, Kang D, et al. PANTHER version 11: expanded annotation data from Gene Ontology and Reactome pathways, and data analysis tool enhancements. Nucleic Acids Res. 2017;45(D1):D183–D9. doi: 10.1093/nar/gkw1138 27899595 PMC5210595

[101] Nishimura D. BioCarta. Biotech Software & Internet Report. 2001;2(3):117–20.

[102] Croft D, Mundo AF, Haw R, Milacic M, Weiser J, Wu G, et al. The Reactome pathway knowledgebase. Nucleic Acids Res. 2014;42:D472–D7. doi: 10.1093/nar/gkt1102 24243840 PMC3965010

[103] Zhang Y. I-TASSER server for protein 3D structure prediction. BMC bioinformatics. 2008;9:40. doi: 10.1186/1471-2105-9-40 18215316 PMC2245901

[104] Berendsen HJC, van der Spoel D, van Drunen R. GROMACS: A message-passing parallel molecular dynamics implementation. Comp Phys Comm. 1995;91:43–56.

[105] Lindahl E, Hess B, van der Spoel D. GROMACS 3.0: A package for molecular simulation and trajectory analysis. J Mol Mod. 2001;7:306–17.

[106] Van Der Spoel D, Lindahl E, Hess B, Groenhof G, Mark AE, Berendsen HJ. GROMACS: fast, flexible, and free. J Comput Chem. 2005;26(16):1701–18. doi: 10.1002/jcc.20291 16211538

[107] Pronk S, Páll S, Schulz R, Larsson P, Bjelkmar P, Apostolov R, et al. GROMACS 4.5: a high-throughput and highly parallel open source molecular simulation toolkit. Bioinformatics. 2013;29(7):845–54. doi: 10.1093/bioinformatics/btt055 23407358 PMC3605599

[108] Lindorff-Larsen K, Piana S, Palmo K, Maragakis P, Klepeis JL, Dror RO, et al. Improved side-chain torsion potentials for the Amber ff99SB protein force field. Proteins. 2010;78(8):1950–8. doi: 10.1002/prot.22711 20408171 PMC2970904

[109] Bussi G, Donadio D, Parrinello M. Canonical sampling through velocity rescaling. J Chem Physics. 2007;126(1):014101. doi: 10.1063/1.2408420 17212484

[110] Berendsen HJC, Postma JPM, van Gunsteren WF, Di Nola A, Haak JR. Molecular dynamics with coupling to an external bath molecular dynamics with coupling to an external bath. J Chem Physics. 1984;81:3684.

[111] Darden T, York D, Pedersen L. Particle mesh Ewald: an N -log (N) method for Ewald sums in large systems. J Chem Physics. 1993;98:10089–92.

[112] Essmann U, Perera L, Berkowitz ML, Darden T, Lee H. A smooth particle mesh Ewald method. J Chem Physics. 1995;103:8577–93.

[113] Hess B, Bekker H, Berendsen HJC, Fraaije JGEM. LINCS: a linear constraint solver for molecular simulations. J Comput Chem. 1998;18:1463–72.

