# Supplementary figures and images for: A whole genome sequencing approach to anterior cruciate ligament rupture–a twin study in two unrelated families
Source: PLoS One. 2022 Oct 6;17(10):e0274354. doi: 10.1371/journal.pone.0274354 (PMC9536556; doi:10.1371/journal.pone.0274354)

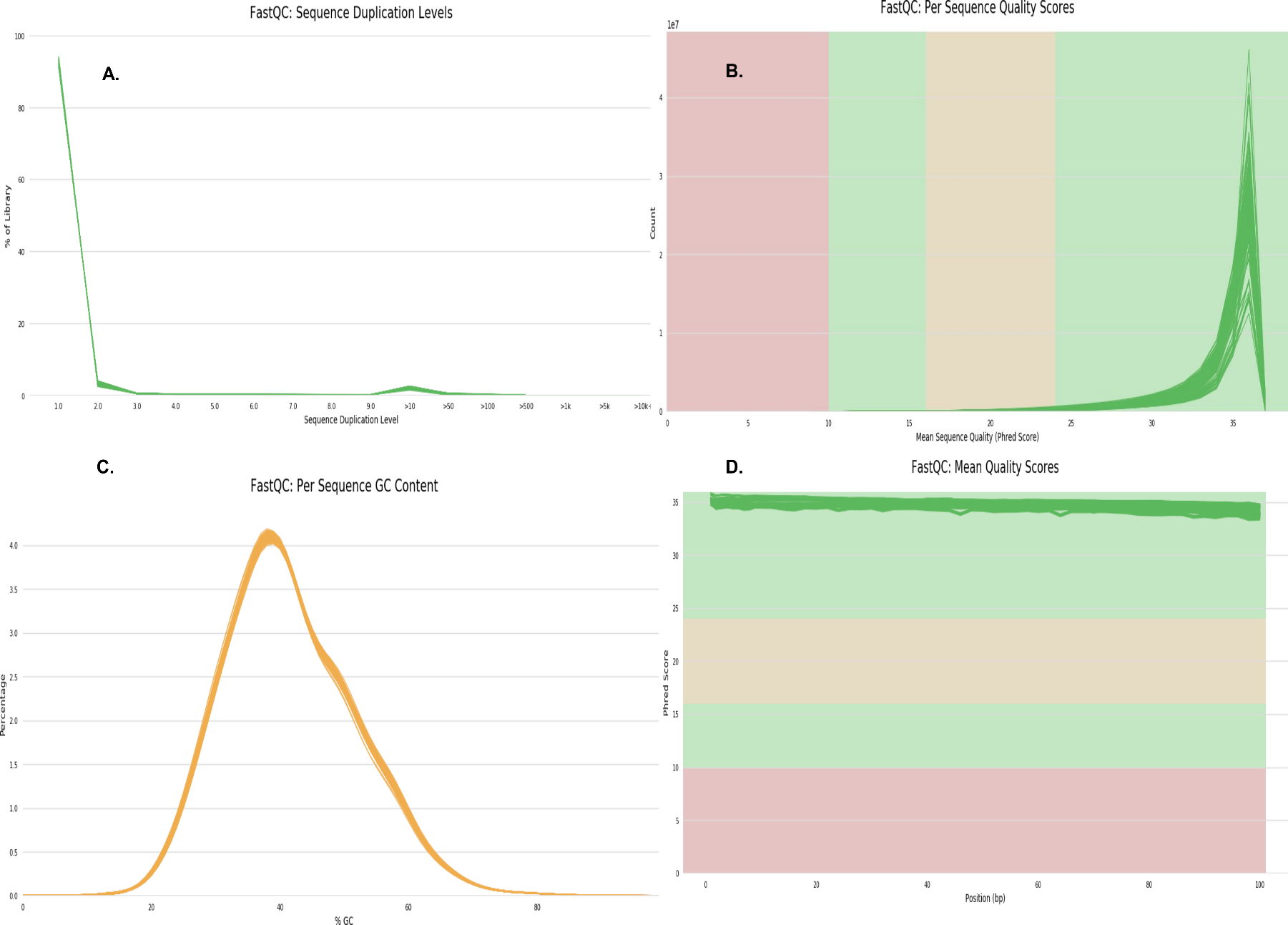

Supplement: S1 Fig — (TIF) [file pone.0274354.s001.tif]

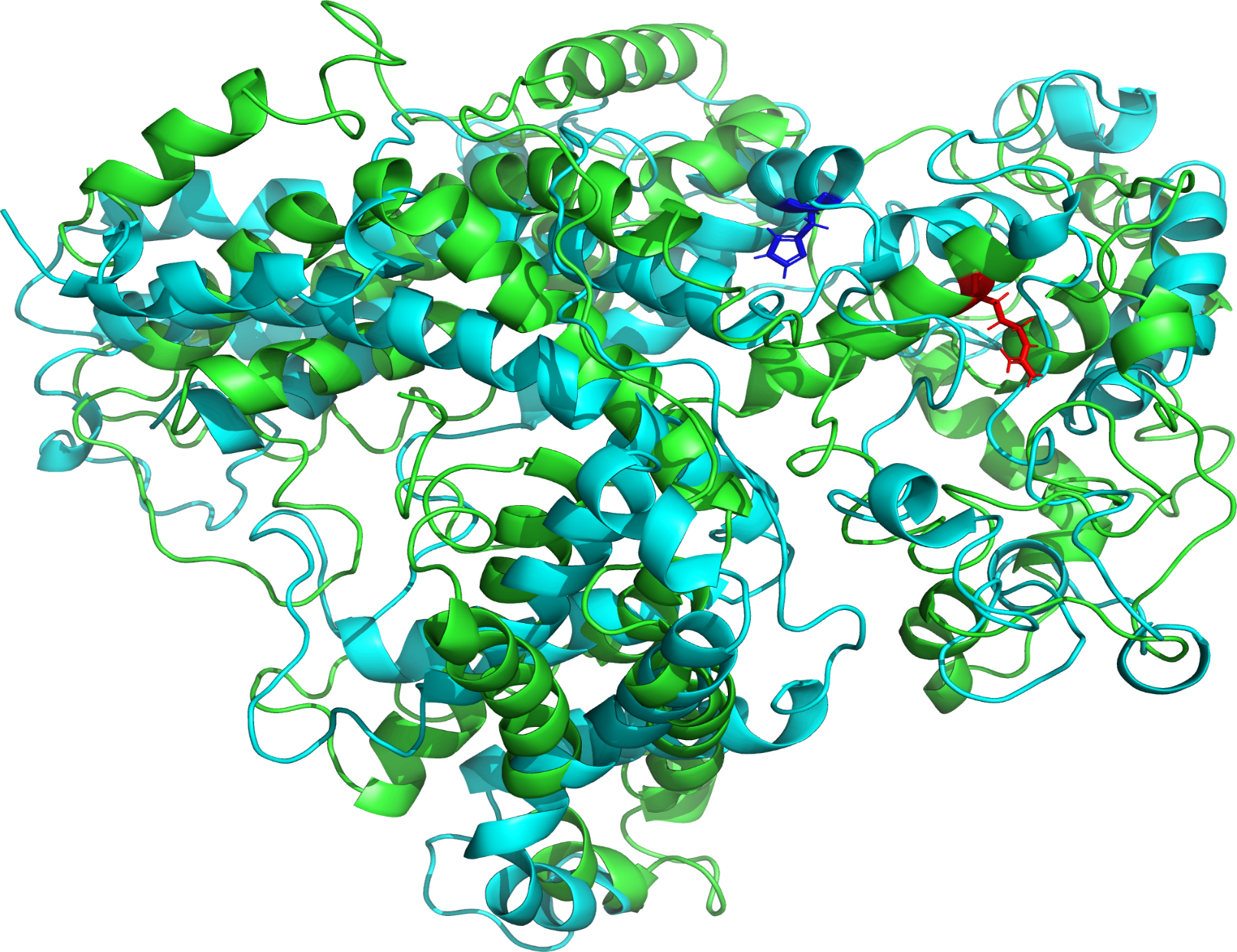

Supplement: S2 Fig — (TIF) [file pone.0274354.s002.tif]

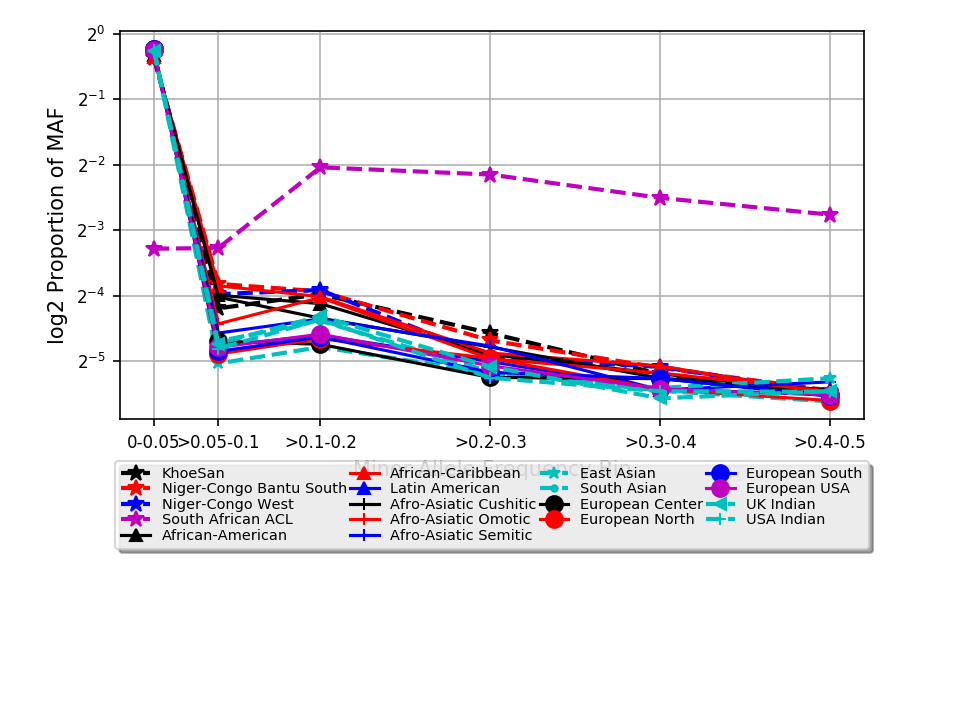

Supplement: S3 Fig — (TIF) [file pone.0274354.s003.tif]

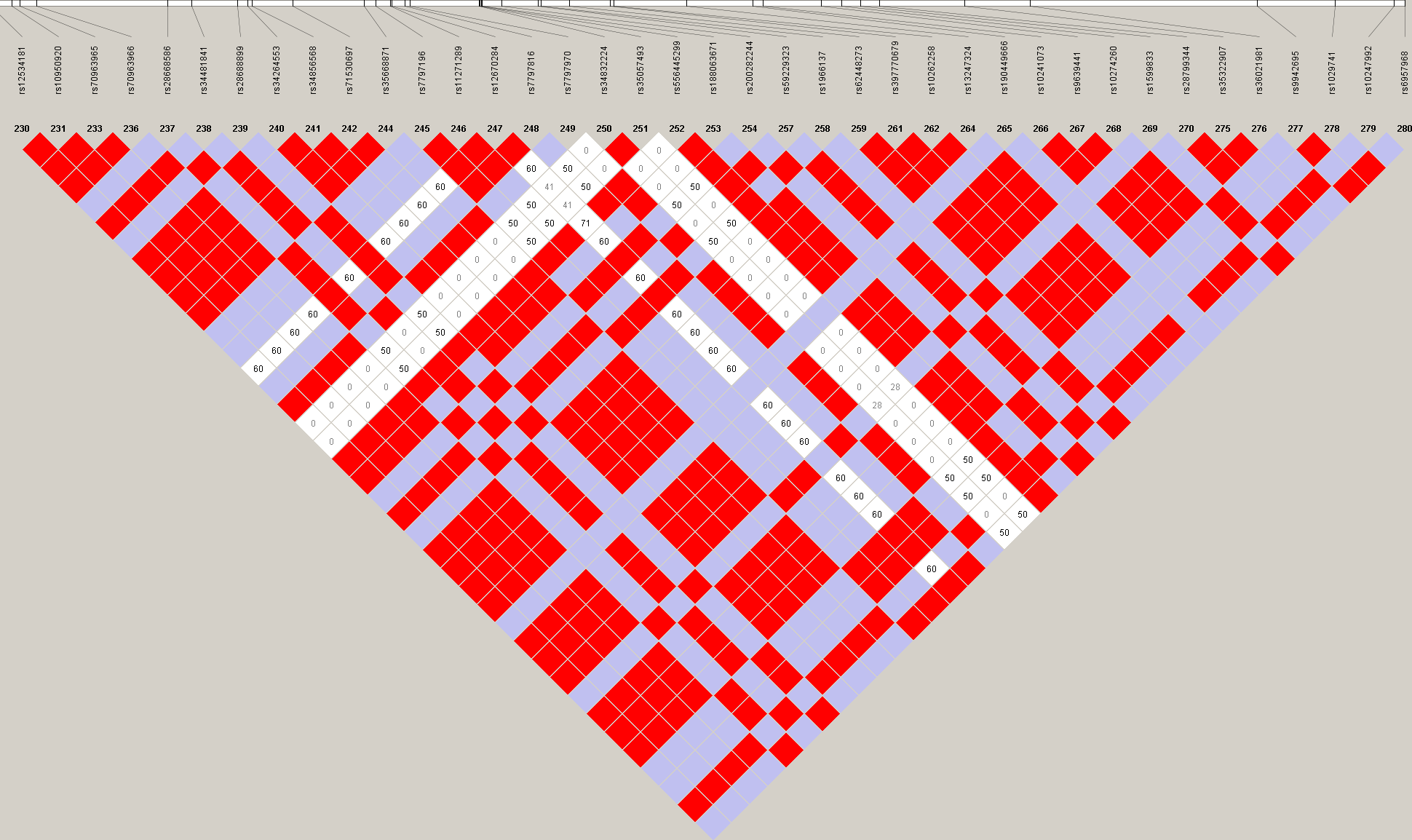

Supplement: S4 Fig — (TIF) [file pone.0274354.s004.tif]
