# Supplementary material for: A whole genome sequencing approach to anterior cruciate ligament rupture–a twin study in two unrelated families
Source: PLoS One. 2022 Oct 6;17(10):e0274354. doi: 10.1371/journal.pone.0274354 (PMC9536556; doi:10.1371/journal.pone.0274354)
Supplement: S1 Table — (DOCX) [file pone.0274354.s005.docx]

**Supplementary Table 1:** History of other musculoskeletal soft tissue injuries in Family A and Family B.

| **Family** | **Family Member** | **History of other injuries (not ACL)** | **Injury Type** |
| --- | --- | --- | --- |
| A | Twin | Yes | Right ankle lateral ligaments and wrist extensor tendon |
|  | Twin | Yes | Right ankle medial ligaments |
|  | Brother | Yes | Left and right tibialis posterior tendon |
|  | Father | No | - |
|  | Mother | No | - |
| B | Twin | Yes | Right ankle medial ligaments |
|  | Twin | Yes | Right ankle lateral ligaments, right shoulder ligaments and right supraspinatus tendon |
|  | Sister | No | - |
|  | Mother | No | - |
