# Supplementary material for: A whole genome sequencing approach to anterior cruciate ligament rupture–a twin study in two unrelated families
Source: PLoS One. 2022 Oct 6;17(10):e0274354. doi: 10.1371/journal.pone.0274354 (PMC9536556; doi:10.1371/journal.pone.0274354)
Supplement: S2 Table — Genes in bold depict predicted pathogenic mutations shared in Family A and B. (DOCX) [file pone.0274354.s006.docx]

**Supplementary Table 2.** Candidate list of genes with predicted mutations in Family A and B.

| **Gene** | **Family A** | **Variant** | **Chromosomal Location** |
| --- | --- | --- | --- |
| *ABCA13* | ATP Binding Cassette Subfamily A Member 13 | rs77147473 | 6p12.3 |
| *AK2* | Adenylate Kinase 2 | rs113711467 | 1p35.1 |
| *AK3* | Adenylate Knase 3 | rs763448502 | 8p24.1 |
| *CAPN2* | Calpain 2 | rs145466296 | 1q41 |
| ***CATSPER2*** | **Cation Channel Sperm Associated 2** | **rs144399798** | **12q15.3** |
| *COL11A1* | Collagen Type XI Alpha 1 Chain | rs139064549 | 1p21.1 |
| ***COL12A1*** | **Collagen Type XII Alpha 1 Chain** | **rs970547** | **6q14.1** |
| *EYS* | Eyes Shut Homolog | rs928941618 | 6q12 |
| *F13A1* | Coagulation factor XIII A chain | rs3024477 | 6p25.1 |
| *F5* | Coagulation Factor V | rs6027 | 1q24.2 |
| *GDF9* | Growth Differentiation Factor 9 | rs61754582 | 3q31.1 |
| *GUCY2C* | Guanylate Cyclase 2C | rs1306674918 | 11p12.3 |
| ***KCNJ12*** | **Potassium Inwardly Rectifying Channel Subfamily J Member 12** | **rs76265595** | **17p11.2** |
|  |  | **rs75029097** |  |
|  |  | **rs77270326** |  |
|  |  | **rs77270326** |  |
| *KIF23* | Kinesin Family Member 23 | rs148511930 | 15q23 |
| *KMT2C* | Lysine Methyltransferase 2C | rs4024419 | 7q36.1 |
| *MASP1* | Mannan Binding Lectin Serine Peptidase 1 | rs140933134 | 3q27.3 |
| *MLH1* | MutL Homolog 1 | rs35001569 | 2p22.2 |
| *MPZL3* | Myelin Protein Zero Like 3 | rs63750449 | 11q23.3 |
| *MYO18A* | Myosin XVIIIA | rs142071163 | 17q11.2 |
| *NLRP14* | NLR Family Pyrin Domain Containing 14 | rs201591312 | 9p15.4 |
| *NPHP4* | Nephrocystin 4 | rs149541731 | 1p36.31 |
| *OVCH2* | Ovochymase 2 | rs35641267 | 11p15.4 |
| *PEX6* | Peroxisomal Biogenesis Factor 6 | rs61759818 | 5p21.1 |
| *PMM1* | Phosphomannomutase 1 | rs577794661 | 17q13.2 |
| *PNKD* | Metallo-Beta-Lactamase Domain Containing | rs750714549 | 1q35 |
| *SI* | Sucrase-Isomaltase | rs147259983 | 3q26.1 |
| *SLC7A2* | Solute Carrier Family 7 Member 2 | rs138434001 | 7p22 |
| *SPG11* | SPG11 Vesicle Trafficking Associated, Spatacsin | rs140440674 | 15q21.1 |
| *TTC38* | Tetratricopeptide Repeat Domain 38 | rs78183930 | 22q13.31 |
| **Gene** | **Family B** | **Variant** | **Chromosomal Location** |
| *ABCC1* | ATP Binding Cassette Subfamily C Member 1 | rs45511401 | 16p13.11 |
| *ABCD4* | ATP Binding Cassette Subfamily D Member 4 | rs45568335 | 14q24.3 |
| *ACAD9* | Acyl-CoA Dehydrogenase Family Member 9 | rs115532916 | 3q21.3 |
| *ATP10A* | ATPase Phospholipid Transporting 10A | rs140982945 | 15q12 |
| ***CATSPER2*** | **Cation Channel Sperm Associated 2** | **rs144399798** | **15q15.3** |
| ***COL12A1*** | **Collagen Type XII Alpha 1 Chain** | **rs970547** | **6q14.1** |
| *DHODH* | Dihydroorotate Dehydrogenase (Quinone) | rs61733129 | 16q22.2 |
| *GKN2* | Gastrokine 2 | rs62133344 | 2p13.3 |
| *GMPR2* | Guanosine Monophosphate Reductase 2 | rs34354104 | 14q12 |
| *HNF4G* | Hepatocyte Nuclear Factor 4 Gamma | unknown | 8q21.13 |
| *INHA* | Inhibin Subunit Alpha - Encodes a member of the TGFB family | rs139051234 | 2q35 |
| *IPP* | Intracisternal A Particle-Promoted Polypeptide | rs142095376 | 1p34.1 |
| ***KCNJ12*** | **Potassium Inwardly Rectifying Channel Subfamily J Member 12** | **rs76265595** | **17p11.2** |
|  |  | **rs75029097** |  |
|  |  | **rs77270326** |  |
|  |  | **rs77270326** |  |
| *PKHD1L1* | PKHD1 Like 1 | rs75029097 | 8q23.1 |
| *PMS2* | PMS1 Homolog 2, Mismatch Repair System Component | rs77270326 | 7p22.1 |
| *RTEL1* | Regulator Of Telomere Elongation Helicase 1 | rs76684759 | 20q13.33 |
| *SEC24D* | SEC24 Homolog D, COPII Coat Complex Component | rs185023340 | 4q26 |
| *SLC26A7* | Solute Carrier Family 26 Member 7 | rs63750123 | 8q21.3 |

Genes in **bold** depict predicted pathogenic mutations shared in Family A and B
