# Supplementary material for: A whole genome sequencing approach to anterior cruciate ligament rupture–a twin study in two unrelated families
Source: PLoS One. 2022 Oct 6;17(10):e0274354. doi: 10.1371/journal.pone.0274354 (PMC9536556; doi:10.1371/journal.pone.0274354)
Supplement: S3 Table — (DOCX) [file pone.0274354.s007.docx]

**Supplementary Table 3.** Genes previously associated with ligament and tendon injury.

| **Gene Name** | **Encoded Protein** | **Chromosomal Location** | **Variant** |
| --- | --- | --- | --- |
| *ACAN* | Aggrecan | 15q26.1 | rs2351491 C/T, rs1042631 C/T, rs1516797 T/G |
| *ADAMTS10* | Metallopeptidase with Thrombospondin Type 1 Motif 10 | 19p13.2 | rs62621197 C/T |
| *ADAMTS17* | Metallopeptidase with Thrombospondin Type 1 Motif 17 | 15q26.3 | rs72755233 G/A |
| *ADIPOQ* | Adiponectin | 3q27.3 | rs1501299 G/T |
| *ANKH* | Progressive ankylosis protein homolog | 5p15.2 | rs3045 A/G |
| *BGN* | Biglycan | Xq28 | rs1126499 C/T, rs1042103 G/A |
| *CASP8* | Caspase-8 | 2q33-q34 | rs3834129 ins/del, rs1045485 G/C |
| *COL1A1* | Pro-α polypeptide of collagen type I | 17q21.33 | rs1107946 G/T |
| *COL3A1* | α1 III collagen chain | 2q31 | rs1800255 G/A |
| *COL5A1* | Pro-α polypeptide of type V collagen | 9q34.2-q34.3 | rs12722 T/C |
| *COL11A1* | α1 XI collagen chain | 1p21 | rs3753841 T/C, rs1676486 C/T |
| *COL11A2* | α2 XI collagen chain | 6p21.3 | rs1799907 T/A |
| *COL12A1* | Pro-α1 polypeptide type XII collagen | 6q12-q13 | rs970547 A/G rs1800012 G/T |
| *COL27A1* | α1 XXVII collagen chain | 9q32 | rs946053 G/T |
| *DCN* | Decorin | 12q22 | rs516115 A/G |
| *DEFB1* | Beta-defensin 1 | 8p23.1 | rs1800972 C/T |
| *EFEMP1* | GF-containing fibulin-like extracellular matrix protein 1 | 2p16.1 | rs3791679 A/G |
| *ESRRB* | Steroid hormone receptor ERR2 | 14q24.3 | rs1676303 C/T |
| *FBN2* | Fibrillin-2 | 5q23-q31 | rs331079 G/T |
| *FGF10* | Fibroblast growth factor 10 | 5p12 | rs11750845 C/T, rs1011814 T/C |
| *FGF3* | Fibroblast growth factor 3 | 11q13.3 | rs12574452 G/A |
| *FGFR1* | Fibroblast growth factor receptor 1 | 8p11.23 | rs13317 T/C |
| *GDF5* | Growth differentiation factor 5 | 20q11 | rs143383 T/C |
| *IL1B* | Interleukin-Iβ | 2q14 | rs16944 T/C |
| *IL1RN* | Interleukin-1 receptor antagonist | 2q14.2 | rs2234663 |
| *IL6* | Interleukin-6 | 1q21 | rs1800795 G/C |
| *IL6R* | Interleukin-6 receptor | 1q21 | rs2221845 A/C |
| *ITGB3* | Integrin beta-3 | 17q21.32 | - |
| *KDR* | Kinase insert domain receptor | 4q11-4q12 | rs2071559 A/G, rs2305948 G/A, rs1870377 T/A |
| *LUM* | Lumican | 12q21.33 | rs2268578 T/C |
| *MIR608* | microRNA 608 | 10q24.31 | rs4919510 C/G |
| *MMP1* | Matrix metalloproteinase 1 | 11q22.3 | rs1799750 1G/2G |
| *MMP3* | Matrix metalloproteinase 3 | 11q22.3 | rs679620 A/G |
| *MMP8* | Matrix metalloproteinase 8 | 11q22.3 | rs11225395 C/T |
| *MMP10* | Matrix metalloproteinase 10 | 11q22.3 | rs486055 C/T |
| *MMP12* | Matrix metalloproteinase 12 | 11q22.3 | rs2276109 A/G |
| *THBS2* | Thrombospondin-2 | 6q27 | rs9406328 C/T |
| *TIMP2* | Metalloproteinase inhibitor 2 | 17q25 | rs4789932 C/T |
| *TNAP* | Tissue-Nonspecific Alkaline Phosphatase | 1p36.12 | rs4654760 C/T |
| *TNC* | Tenascin-C glycoprotein | 9q33 | rs1330363 A/G, rs2104772 T/A, rs13321 G/C  rs1138545 G/A, rs3789870 C/T, rs7021589 A/G  rs10759753 T/C, rs72758637 G/C, rs7035322 G/T |
| *VEGFA* | Vascular endothelial growth factor A | 6p21.1 | rs699947 C/C, rs1570360 G/A, rs2010963 G/C |
