# Supplementary material for: A whole genome sequencing approach to anterior cruciate ligament rupture–a twin study in two unrelated families
Source: PLoS One. 2022 Oct 6;17(10):e0274354. doi: 10.1371/journal.pone.0274354 (PMC9536556; doi:10.1371/journal.pone.0274354)
Supplement: S4 Table — (DOCX) [file pone.0274354.s008.docx]

**Supplementary Table 4**. The table displays the top significant pathways, GO biological process, Molecular Function and Human Phenotypes associated with the genes previously associated with tendon and ligament injury, and the candidate list of predicted pathogenic genes and their interacting genes for Family A and Family B combined and independently.

| **Enrichment** | **P-value** | **Adjusted P-value** | **Database** |
| --- | --- | --- | --- |
| **Pathway** | | | |
| **Genes Previously Associated with Tendon and Ligament Injury** |  |  |  |
| PI3K-Akt signalling pathway | 8.9e-11 | 2.8e-8 | KEGG-Human 2019 |
| Protein digestion and absorption | 5.9e-10 | 9.0e-8 |  |
| Rheumatoid arthritis | 1.1e-6 | 1.0e-4 |  |
| Extracellular matrix organization | 1.9e-32 | 2.9e-29 | Reactome 2016 |
| Degradation of the extracellular matrix | 7.1e-13 | 5.4e-10 |  |
| Collagen formation | 6.4e-12 | 3.3e-9 |  |
| Assembly of collagen fibrils and other multimeric structures | 1.4e-11 | 5.6e-9 |  |
| Integrin signalling pathway | 2.7e-8 | 3.1e-6 | Panther 2016 |
| **Family A and B** |  |  |  |
| Complement and coagulation cascades | 3.0e-7 | 1.0e-4 | KEGG-Human 2019 |
| Purine metabolism | 6.0e-7 | 9.2e-4 |  |
| Mismatch repair | 6.9e-5 | 7.1e-3 |  |
| Lectin Induced Complement Pathway | 1.2e-5 | 2.7e-3 | Biocarta 2016 |
| Metabolism of nucleotides | 1.0e-5 | 9.1e-4 | Reactome 2016 |
| Lectin pathway of complement activation | 1.0e-5 | 6.2e-4 |  |
| Common Pathway of Fibrin Clot Formation | 6.0e-5 | 3.1e-2 |  |
| **Family A** |  |  |  |
| Complement and coagulation cascades | 3.0e-9 | 9.0e-7 | KEGG-Human 2019 |
| Mismatch repair | 3.9e-5 | 3.0e-3 |  |
| **Family B** |  |  |  |
| ABC transporters | 8.0e-10 | 2.5e-7 | KEGG-Human 2019 |
| Mismatch repair | 2.1e-7 | 3.2e-5 |  |
| **Go Biological Processes** | | | |
| **Genes Previously Associated with Tendon and Ligament Injury** |  |  |  |
| Extracellular matrix organization | 1.5e-28 | 7.8e-25 | GO Biological Process 2018 |
| Extracellular matrix disassembly | 4.6e-14 | 1.1e-10 |  |
| Collagen fibril organization | 1.7e-13 | 2.9e-10 |  |
| **Family A and B** |  |  |  |
| Establishment of protein localization to peroxisome | 1.4e-6 | 7.3e-3 | GO Biological Process 2018 |
| Peroxisomal transport | 3.0e-6 | 5.7e-3 |  |
| Complement activation, lectin pathway | 8.0e-6 | 8.2e-3 |  |
| Mismatch repair | 2.0e-6 | 6.7e-3 |  |
| **Family A** |  |  |  |
| Establishment of protein localization to peroxisome | 8.0e-7 | 4.1e-3 | GO Biological Process 2018 |
| Peroxisomal transport | 1.3e-6 | 3.3e-3 |  |
| Nucleobase-containing small molecule interconversion | 2.2e-6 | 3.7e-3 |  |
| **Family B** |  |  |  |
| Cargo loading into COPII-coated vesicle | 1.2e-8 | 6.1e-5 | GO Biological Process 2018 |
| Antigen processing and presentation of peptide antigen via MHC class I | 5.6e-7 | 9.4e-4 |  |
| Mismatch repair | 9.5e-7 | 1.2e-3 |  |
| **Molecular Function** | | | |
| **Genes Previously Associated with Tendon and Ligament Injury** |  |  |  |
| Growth factor receptor binding | 7.3e-10 | 8.5e-7 | GO Molecular Function 2018 |
| Metalloendopeptidase activity | 2.2e-7 | 1.3e-4 |  |
| Integrin binding | 1.4e-6 | 4.1e-4 |  |
| Growth factor receptor binding | 7.3e-10 | 8.5e-7 |  |
| **Family A and B** |  |  |  |
| Adenylate kinase activity | 1.4e-6 | 1.6e-3 | GO Molecular Function 2018 |
| **Family A** |  |  |  |
| Adenylate kinase activity | 2.0e-9 | 2.5e-6 | GO Molecular Function 2018 |
| Nucleotide kinase activity | 5.0e-7 | 3.1e-4 |  |
| Satellite DNA binding | 1.7e-4 | 2.5e-2 |  |
| Histone methyltransferase activity | 1.3e-5 | 2.9e-3 |  |
| **Family B** |  |  |  |
| ATPase-coupled anion transmembrane transporter | 1.2e-8 | 2.7e-6 | GO Molecular Function 2018 |
| **Human Phenotype** | | | |
| **Genes Previously Associated with Tendon and Ligament Injury** |  |  |  |
| Osteoarthritis | 2.2e-12 | 4.0e-9 | Human Phenotype Ontology |
| **Family A and B** |  |  |  |
| Autosomal recessive inheritance | 5.0e-10 | 8.1e-8 | Human Phenotype Ontology |
| **Family A** |  |  |  |
| Abnormality of the common coagulation pathway | 1.5e-6 | 8.7e-5 | Human Phenotype Ontology |
| Autosomal recessive inheritance | 1.9e-7 | 8.4e-5 |  |
| **Family B** |  |  |  |
| Autosomal recessive inheritance | 6.9e-7 | 1.2e-4 | Human Phenotype Ontology |
