# Supplementary material for: A whole genome sequencing approach to anterior cruciate ligament rupture–a twin study in two unrelated families
Source: PLoS One. 2022 Oct 6;17(10):e0274354. doi: 10.1371/journal.pone.0274354 (PMC9536556; doi:10.1371/journal.pone.0274354)
Supplement: S5 Table — Genes in bold script: highlighted as previously associated with musculoskeletal injury susceptibility. (DOCX) [file pone.0274354.s009.docx]

**Supplementary Table 5.** Inferred functional partners and enriched pathways for genes of interest in Family A and B.

| **GENE OF INTEREST** | **TOP 100 INFERRED FUNCTIONAL PARTNERS** | **ENRICHMENT PATHWAYS** | **SUMMARY OF INFERRED PARTNERS** |
| --- | --- | --- | --- |
| *COL11A1*  rs139064549 (G/C) | *BMP1,BRAF,CAPN2,CDC42,COL10A1,****COL11A2,COL12A1****,COL13A1,COL14A1,COL15A1,COL16A1,COL17A1,COL18A1,COL19A1,****COL1A1****,COL1A2,COL20A1,COL21A1,COL22A1,COL23A1,COL24A1,COL25A1,****COL27A1,****COL2A1,COL3A1,COL4A1,COL4A2,COL4A3,COL4A4,COL4A5,COL4A6,****COL5A1****,COL5A2,COL5A3,COL6A1,COL6A2,COL6A3,COL7A1,COL8A1,COL8A2,COL9A1,COL9A2, COL9A3,EGFR,ELN,FBN1,****FBN2****,FN1,GRB2,ITGA1,ITGA10,ITGA11,ITGA2,ITGA2B,ITGA3,ITGA4,ITGA5,ITGA6,ITGA7,ITGA8,ITGA9,ITGAD,ITGAE,ITGAL,ITGAM,ITGAV,ITGAX,ITGB1,ITGB2,****ITGB3****,ITGB4,ITGB5,ITGB6,ITGB7,ITGB8,****KDR****,KRAS,LAMA1,LAMA2,LAMA3,LAMA4,LAMA5,LAMB1,LAMB2,LAMB3,LAMC1,LAMC2,LAMC3,MAPK1,MAPK3,PDGFA,PRKCA,PXN,RHOA,SPP1,****TGFB1****,TGFB2,TGFB3,****TNC****,VCAN* | - Regulation of the actin cytoskeleton - Focal adhesion - PI3K-Akt signalling pathway - ECM organization | Most of the genes (39%) were collages, with integrin and laminin encoding genes making up 26 and 11% of the genes respectively. Proteoglycans made-up 2% of the genes, with 5% of the genes encoding for growth factors. 4% were glycoprotein encoding genes and 3% protein kinase encoding genes. |
| *COL12A1*  rs970547 (C/T) | *HSPG2,****ACAN****,ACTA1,AKT1,****BGN****,BMP1,BMP2,BMP4,BRAF,CAPN3,CASP3,COL10A1,****COL11A1,COL11A2****,COL13A1,COL14A1,COL15A1,COL17A1,COL18A1,COL19A1,****COL1A1****,COL1A2,COL21A1,COL22A1,COL23A1,COL24A1,COL25A1,****COL27A1****,COL2A1,COL3A1,COL4A1,COL4A2,COL4A3,COL4A4,COL4A5,****COL5A1****,COL5A2,COL5A3,COL6A1,COL6A2,COL6A3,COL7A1,COL8A1,COL8A2, COL9A1,COL9A2,COL9A3,CREB1,DAG1,****DCN****,DMD,****EFEMP1****,EFEMP2,EGFR,ELN,FBN1,****FBN2****,FGFR3,FN1,****GDF5****,GNAS,HAPLN1,IGF1,ITGA11,ITGA2B,ITGA3,ITGA5,ITGA6,ITGA7,ITGAV,ITGB1,ITGB2,****ITGB3****,ITGB4,****KDR****,KRAS,LAMA1,LAMA2,LAMA3,LAMA4,LAMA5,LAMB1,LAMB2,LAMB3,LAMC1,LAMC2,LMNA,LTBP4,MMP13, MMP9,PDPK1,PTEN,PTK2,PP1,SRC,****TGFB1****,TGFB2,TGFB3,TIMP1,VCAN* | - ECM-receptor interaction - Focal adhesion - ECM organization - Collagen formation - Collagen biosynthesis and modifying enzymes - Assembly of collagen fibrils - ECM proteoglycans | 36% of the genes code for collagens with 11% for both integrin and laminin encoding proteins. Proteoglycan encoding genes made-up 7%, with growth factors contributing 9% of the genes. Genes encoding protein kinases and glycoproteins both made-up 5% of the genes. |
| *CATSPER2*  rs144399798 (C/T) | *ACR,ADAM2,ADAM20,ADAM21,ADAM30,ADCY10,AURKB,B4GALT1,B4GALT7,BRCA1,BRSK2,CACNA1C,CACNA1D,CACNA1F,CACNA1S,CACNB4,CACNG7,CALM2,CATSPER1,CATSPER3,CATSPERB,CATSPERD,CATSPERG,CD9,CNGA3,FOS,GABRA1,GABRA4,GABRG3,GNA14,GNAS,GNAZ,GNB1,GNB4,GNG7,GNRH1, HCN2,HVCN1,ITPR1,IZUMO1,IZUMO2,IZUMO4,JUN,KCNK10,KCNU1,KCNV2,KCTD3,KCTD6,MAP2K1,MAP2K3,MAP2K4,MAP3K11,MAPK3,MAPK8,MAPK9,MERTK,MYH14,MYH2,MYH9,MYO1C, MYO6,MYO7A,MYO9B,NOS1,NOS3,NPR1,OVGP1,PDE4B,PLA2G10,PLA2G2A,PLA2G4A,PLA2G6,PLCD1,PLCD4,PLCZ1,PPP1R12A,PRKAR1A,PRKCE,PRKCQ,PRKG1,PTK2,RORA,RORB,SLC22A3,SPAM1,STK11,STK33,STK38,STK39,STRC,TAS2R16,TAS2R7, TET1,TSSK6,TXK,ZAN,ZP1,ZP2,ZP3ZP4* | - Gonadotropin-releasing hormone signalling pathway - Fertilization - Reproduction pathways | 20% of the genes are involved in fertilization pathways, 19% of the genes encode for protein kinases, and the remaining are G-proteins, transmembrane proteins, glycoproteins, and proteins involved in calcium signalling and channel activity. |
| *KCNJ12*  rs76265595 (G/A)  rs75029097 (G/A)  rs77270326 (G/A) | *ABCC8,ABCC9,ADCY1,ADCY2,ADCY3,ADCY4,ADCY5,ADCY6,ADCY7,ADCY8,ADCY9,AKAP9,ATP2A2,ATP2A3,ATP2C1,ATP6AP1,ATP6AP2,ATP6V0D1,B2M,CACNA1C,CACNA1D,CACNA1F,CACNA1S,CACNA2D1,CACNB1,CACNB2,CACNB3,CACNB4,CACNG4,CACNG7,CALM1,CALM2,CALM3,CAMK2B,CAMK2D,CAMK4,CAV3,CYP17A1,GABRA6,GNAI1,GNAI2,GNAI3,GNAS,GNB1,GNB2,GNB3,GNG10,GNG12,GNG2,GNG3,GNG4,GNG5,GNG7,GNG8,GNGT1,GNGT2,GUCY1A1,GUCY1A2,HCN4,HSPD1,ITPR1,ITPR2,JUN,KCNA1,KCNA5,KCND2,KCND3,KCNE3,KCNG2,KCNG4,KCNH2,KCNH4,KCNJ1,KCNJ10,KCNJ11,KCNJ14,KCNJ15,KCNJ16,KCNJ2,KCNJ3,KCNJ4,KCNJ5,KCNJ6,KCNJ8KCNJ9,KCNK3,KCNMB1,KCNQ1,KCNQ2,KCNQ3,KRAS,MAPK1,PLCB3,PRKACA,PRKACB,PRKCA,RYR1,RYR2,SCN5A,VCL* | - Circadian entrainment - Cholinergic synapse - Oxytocin signalling pathway - GABAergic synapse - Morphine addiction - Inwardly rectifying K+ channels - Activation of G protein gated K+ channels | The majority of the genes encode for proteins involved in ion channel activity (46%). Other genes encode for G proteins (17%) and adenylate cyclase’s (9%). |
| *GP6* | *AKT1,AKT2,AKT3,BSG,CD36,CD40LG,CD44,CD47,CDKN2A,CLEC1B,****COL1A1****,COL1A2,COL4A1,COL4A2,CSF2,EDIL3,F2,F2R,F3, F5,F8,F9,FCER1G,FGA,FGB,FGG,FN1,FYN,GATA3,GNA13,GNAQ,GNAS,GP1BA,GP1BB,GP5,GP9,HDAC2,IFNAR1,IGF2,IL2,IL4,****IL6****,IL6ST,ITGA1,ITGA2,ITGA2B,ITGA3,ITGAV,ITGB1,ITGB2,****ITGB3****, JAK2,JAM3,KIT,KRAS,LUM,LYN,MAPK1,MERTK,NCOA3,NOS3, NRAS,PDPK1,PIK3CA,PIK3CB,PIK3CG,PIK3R1,PLA2G4A,PLAT, PLAU,PLCG2,PLG,PRKCZ,PROC,PROS1,PSEN1,PTGIR,PTGS1,PTPN11,PTPN6,SDC1,SELE,SELL,SELP,SERPINC1,SERPINE1,SHC1,SLC7A11,SNAI2,SOD1,SPP1,SRC,STIM1,****TGFB1****,THBD,THBS1,TNF,TP53,****VEGFA****,VWF* | - ECM-receptor interaction and organization - Hemostasis - Cell surface interactions at the vascular wall | Collagen genes comprised only 4%, while glycoproteins and protein kinases made-up 12 and 10% of the protein encoding genes respectively. Further, integrins (8%) and interleukins (4%) contributed to the functional genes. The remaining genes coded for coagulation factors, fibrinogens, proteoglycans and growth factors. |
| *MIR99A* | *APC,BRCA1,CASP3,CCND1,CD44,CDKN1A,CDKN1B,CDKN2A,EGFR,ERBB2,HRAS,KRAS,MAPK1,MIR100,MIR101.1,MIR103A1,MIR106B,MIR10A,MIR10B,MIR122,MIR124.1,MIR125A,MIR125B1,MIR126,MIR129.1,MIR135A1,MIR135B,MIR141,MIR143,MIR145,MIR146A,MIR150,MIR155,MIR15A,MIR15B,MIR17,MIR181A1,MIR183,MIR18A,MIR192,MIR193B,MIR195,MIR199A1,MIR19A,MIR200A, MIR200B,MIR200C,MIR205,MIR20A,MIR21,MIR210,MIR214,MIR221,MIR222,MIR223,MIR224,MIR23A,MIR23B,MIR25,MIR26B,MIR27A,MIR27B,MIR28,MIR29A,MIR29B1,MIR29C,MIR30A,MIR30B,MIR30C1,MIR30E,MIR31,MIR324,MIR330,MIR331,MIR335,MIR342,MIR34A,MIR34C,MIR373,MIR423,MIR483,MIR91,MIR92A1,MIR96,MIRLET7A1,MIRLET7B,MIRLET7D,MIRLET7E,MIRLET7G,MIRLET7I, MMP9,MTOR,MYC,NOTCH1,PIK3CA,PTEN,PTGS2,STAT3,TP5,* ***VEGFA*** | - MicroRNAs in cancer - Proteoglycans in cancer | 75% of the inferred genes encoded for miRNAs. The remaining genes encode for glycoproteins, protein kinases and growth factors. |
| *MIR99AHG* | *AANAT,ABCB1,ABL1,ADGRG4,ADPGK,AKT1,ALX3,ANKRD1,APAF1,APOL6,ARHGAP20,ASB2,ASXL1,BAALC,BAD,BAX,BCL2L1,BCR,CBFA2T2,CBFB,CBL,CCNA1,CCNA2,CD14,CD33,CD34,CDK4, CDKN1A,CEBPA,CHIC2,CHID1,CREBBP,CSF1R,CSF2,CSF3,CSF3R,DNMT3A,ELAVL3,ELF4,ERG,ETV6,FES,FLT3,FUS,GATA2,HOXA9,HRAS,IDH1,IDH2,IL3,ITGAM,JAK2,KAT6A,KIT,KMT2A,KRAS,LIF,LYN,MAML3,MAP2K1,MAPK1,MCL1,MECOM,MEIS1,MIR100HG,MLF1,MLF2,MNDA,MPO,MYC,MYH11,NPM1,NRAS,NSD1,NTRK2,NUP214,NUP98,PCM1,PICALM,PIM1,PRDM16,PTPN11,RAF1,RARA,RUNX1,RUNX1T1,RUNX2,RUNX3,SEM1,SNAP91,SPI1,STAT3,STAT5A,STAT5B,TERT,TET2,****TGFB1****,TP53,U2AF1,WT1* | - VEGF signalling pathway | Transcription factors and co-activators made-up 23% of the protein encoding genes, with 14% encoding for protein kinases. Apoptosis related genes were encoded by 5% of the genes. |
| *MIR125B2* | *MIR125B1,ABCB1,ABCC1,AKT1,AKT2,APC,BAK1,BCL2,BRCA1, CASP3,CCND1,CCNE1,CD44,CDK6,CDKN1A,CDKN1B,CDKN2A,CTNNB1,DNMT1,DNMT3B,E2F1,E2F2,E2F3,EGF,EGFR,ERBB2,FGF2,HRAS,IGF1,IGF1R,IKBKB,MAP2K1,MAP2K2,MAPK1,MAPK3,MAPK8,MDM2,MIR103A2,MIR10B,MIR1.1,MIR125A,MIR126,MIR128.2,MIR130B,MIR133B,MIR137,MIR140,MIR155,MIR15B,MIR181A2,MIR181C,MIR185,MIR195,MIR197,MIR199A1,MIR199A2,MIR199B,MIR21,MIR221,MIR222,MIR23A,MIR23B,MIR25,MIR27B,MIR30E,MIR34A,MIR451A,MIR483,MIR494,MIR73,MIR92,MIR96,MIR99A,MIRLET7C,MTOR,MYEF2,NFKB1,NRAS,PDGFRA,PIK3CA,PIK3CB,PIK3CD,PIK3CG,PIK3R1,PIK3R2,PIK3R3,PPP3R1,PRKCA,PRKCB,PTEN,RAF1,RASGRF1,RHOA,ROCK1,STAT3,****TGFB1****,TNF,TP53,****VEGFA****,WNT3A* | - MiRNAs in cancer - PI3K-Akt signalling pathway | Most of the genes (38%) encoded for miRNAs, with 18 and 5% encoding for protein kinases and growth factors respectively. The remaining genes encoded transcription factors, hormones, angiogenesis related genes. |
| *MIRLET7C* | *ABCB1,ABCC1,APC,ATM,BCL2,BCL2L11,BRCA1,CASP3,CCND1,CD44,CDC42,CDK6,CDKN1A,CDKN1B,CDKN2A,DNMT1,DNMT3B,E2F1,E2F3,EGFR,ERBB2,EZH2,HDAC1,HRAS,KRAS,MAP2K1,MAPK1,MAPK3,MCL1,MDM2,MIR100,MIR101.1,MIR101.2,MIR106B,MIR125A,MIR126,MIR128.2,MIR143,MIR145,MIR146A,MIR148A, MIR148B,MIR150,MIR155,MIR15A,MIR16.1,MIR17,MIR181A1,MIR183,MIR195,MIR199A1,MIR199B,MIR200A,MIR200B,MIR200C,MIR203A,MIR21,MIR210,MIR221,MIR222,MIR223,MIR23A,MIR23B, MIR27A,MIR27B,MIR29A,MIR29B1,MIR29B2,MIR29C,MIR30A,MIR31,MIR335,MIR34A,MIR34B,MIR34C,MIR451A,MIR4763,MIR9.1,MIR99A,MIRLET7A1,MIRLET7A2,MIRLET7A3,MIRLET7B,MIRLET7BHG,MIRLET7D,MIRLET7E,MIRLET7F1,MIRLET7F2,MIRLET7G, MMP9,MTOR,MYC,NOTCH1,PIK3CA,PIK3R1,PTEN,SIRT1,STAT3,TP53,****VEGFA*** | - Angiogenesis - VEGF signalling pathway - Interleukin signalling pathway | Most of the genes (59%) encode for miRNAs. The remaining genes encode for glycoproteins, protein kinases and growth factors. Furthermore, a few of the genes are involved in apoptotic pathways. |
| *LINC01250* | *no data found* | · |  |

Genes in **bold** script: highlighted as previously associated with musculoskeletal injury susceptibility.
