# Supplementary material for: A whole genome sequencing approach to anterior cruciate ligament rupture–a twin study in two unrelated families
Source: PLoS One. 2022 Oct 6;17(10):e0274354. doi: 10.1371/journal.pone.0274354 (PMC9536556; doi:10.1371/journal.pone.0274354)
Supplement: S1 File — (PDF) [file pone.0274354.s011.pdf]

UNIVERSITY OF CAPE TOWN
Faculty of Health Sciences
Human Research Ethics Committee

Room E53-46 Old Main Building
Groote Schuur Hospital
Observatory 7925
Telephone [021] 406 6626
Email: shuretta.thomas@uct.ac.za
Website: www.health.uct.ac.za/fhs/research/humanethics/forms

04 December 2017

HREC REF: 823/2017

A/Prof Alison September
Human Biology
Sport Science Institute

Dear A/Prof September

PROJECT TITLE: WHOLE GENOME SEQUENCING APPROACH TO IDENTIFYING GENETIC RISK FACTORS UNDERLYING ANTERIOR CRUCIATE LIGAMENT INJURIES IN A TWIN FAMILY STUDY (PhD-candidate- Ms D Feldmann)

Thank you for submitting your study to the Faculty of Health Sciences Human Research Ethics Committee.

It is a pleasure to inform you that the HREC has **formally approved** the above-mentioned study.

Approval is granted for one year until the 30 December 2018.

Please submit a progress form, using the standardised Annual Report Form if the study continues beyond the approval period. Please submit a Standard Closure form if the study is completed within the approval period.
(Forms can be found on our website: www.health.uct.ac.za/fhs/research/humanethics/forms)

Please quote the HREC REF in all your correspondence.

Please note that the ongoing ethical conduct of the study remains the responsibility of the principal investigator.

Please note that for all studies approved by the HREC, the principal investigator **must** obtain appropriate institutional approval, where necessary, before the research may occur.

The HREC acknowledge that the student, Daneil Feldmann will also be involved in this study.

Yours sincerely

PROFESSOR M BLOCKMAN
CHAIRPERSON, FHS HUMAN RESEARCH ETHICS COMMITTEE
Federal Wide Assurance Number: FWA00001637.
Institutional Review Board (IRB) number: IRB00001938

This serves to confirm that the University of Cape Town Human Research Ethics Committee complies to the Ethics Standards for Clinical Research with a new drug in patients, based on the Medical Research Council (MRC-SA), Food and Drug Administration (FDA-USA), International Convention on Harmonisation Good Clinical Practice (ICH GCP), South African Good Clinical Practice Guidelines (DoH 2006), based on the Association of the British Pharmaceutical Industry Guidelines (ABPI),and Declaration of Helsinki (2013) guidelines.
The Human Research Ethics Committee granting this approval is in compliance with the ICH Harmonised Tripartite Guidelines E6: Note for Guidance on Good Clinical Practice (CPMP/ICH/135/95) and FDA Code Federal Regulation Part 50, 56 and 312.
